# Transdiagnostic types of formal thought disorder and their association with gray matter brain structure: a model-based cluster analytic approach

**DOI:** 10.1038/s41380-025-03009-w

**Published:** 2025-04-11

**Authors:** Frederike Stein, Anna Merle Gudjons, Katharina Brosch, Luca Mira Keunecke, Julia-Katharina Pfarr, Lea Teutenberg, Florian Thomas-Odenthal, Paula Usemann, Hanna Wersching, Adrian Wroblewski, Kira Flinkenflügel, Janik Goltermann, Dominik Grotegerd, Susanne Meinert, Katharina Thiel, Alexandra Winter, Nina Alexander, Tim Hahn, Hamidreza Jamalabadi, Andreas Jansen, Axel Krug, Igor Nenadić, Benjamin Straube, Udo Dannlowski, Tilo Kircher

**Affiliations:** 1 Department of Psychiatry and Psychotherapy, University of Marburg, Marburg, Germany; 2 Center for Mind, Brain and Behavior, University of Marburg, Marburg, Germany; 3 Institute of Behavioral Science, Feinstein Institutes for Medical Research, Manhasset, NY USA; 4 Institute for Translational Psychiatry, University of Münster, Münster, Germany; 5 Institute for Translational Neuroscience, University of Münster, Münster, Germany; 6 Core-Facility Brainimaging, Faculty of Medicine, University of Marburg, Marburg, Germany; 7 Department of Psychiatry und Psychotherapy, University Hospital, Bonn, Germany

**Keywords:** Neuroscience, Psychiatric disorders

## Abstract

Formal thought disorder (FTD) is a complex syndrome affecting language and thought processes in psychotic and affective disorders. Clustering (i.e., identification of data-driven clinical subtypes) establishes latent (sub-) structures into psychopathological syndromes. A latent profile analysis (LPA) of FTD symptoms was conducted in 1 032 patients diagnosed with Schizophrenia-Spectrum-Disorders (n = 107), Major Depressive (n = 800), and Bipolar Disorder (n = 125). Clusters were compared for cognition and psychopathology. Associations with gray matter volume (GMV) and cortical surface (gyrification, cortical complexity, sucal depth) were explored using T1-weighted MRI data, analyzed with CAT12. Robustness-analyses in an age- and sex-matched subsample (n = 321) with the same n for each diagnosis (n = 107) were applied. LPA revealed 4 transdiagnostic clusters: *minimal* FTD, *poverty*, *inhibition*, *severe* FTD that remained stable in an age- and sex-matched subsample and in each diagnosis separately. Patients exhibiting *severe* FTD compared to *minimal* FTD showed GMV reductions in the right superior and middle frontal gyri. *Inhibition* showed a GMV reduction in the right inferior and middle temporal gyri, and fusiform gyrus compared with *minimal* and *severe* FTD. Sulcal depth was reduced around the left insula, superior temporal sulcus and temporal pole in the *poverty* cluster, and in the bilateral insula in the *severe* cluster, both compared to the *inhibition* cluster. No results for cortical thickness, gyrification, and complexity were found. Results from the total sample could be replicated in the matched subsample. Our results unravel the clinical heterogeneity of FTD psychopathology across affective and psychotic disorders. Associations of FTD clusters with neuroanatomical substrates imply language-related brain structures being involved in thought and language impairment.

## Introduction

Impairments in speech production and aberrations in the form of thought are clinically termed formal thought disorder (FTD). FTD is a core feature of Schizophrenia (SZ) but also occurs in other disorders including Schizoaffective Disorder (SZA), Major Depressive Disorder (MDD), or Bipolar Disorder (BD) [1–7]. The presence of FTD is considered as a marker of illness severity, FTD can predict new episodes, and (re-) hospitalization is both more likely and significantly longer for patients with FTD [8]. Moreover, it is associated with poor social functioning [9] and impacts the perceived quality of life in SZ and SZA patients (henceforth referred to as schizophrenia-spectrum-disorders, SSD) [10]. Of importance is the widely unacknowledged fact, that the prevalence of FTD in MDD ranges from 36 to 53%, in SZ from 50 to 81%, and in SZA up to 60% [8]. Moreover, a meta-analysis reported no quantitative difference of FTD in acute BD and SZ [2].

FTD is a multifaceted syndrome. Studies examining the factorial structure of FTD symptomatology in SZ have identified factors ranging from two [2, 11] to six positive/disorganized factors [12], while one negative domain was consistently identified [11]. Positive FTD factors mostly include symptoms such as derailment and pressure of speech. In contrast, the negative domain can be characterized by a decrease in the amount of produced speech [3, 13]. Yet, research on both FTD symptomatology and underlying neurobiological mechanisms was almost exclusively performed in SZ. We have investigated transdiagnostic factors of FTD across affective and psychotic disorders [6, 7], lately using FTD symptoms from the Scales for the Assessment of Positive and Negative Symptoms (SAPS; SANS) [14, 15]. Hereof, three factors (i.e., disorganization, emptiness, incoherence) were delineated across MDD, BD, and SSD patients [7]. This factor solution was replicated using a different FTD rating scale [16].

Lately, there has been an increasing interest in data-driven and statistical tools such as clustering techniques and finite mixture modelling to assess clinical heterogeneity in psychiatric disorders with regard to symptoms, clinical course and underlying biological mechanisms [17]. In contrast to the above mentioned FTD symptom factor analyses, unsupervised clustering attempts to group patients based on indicator variables whereas factor analyses group a set of variables, in this case symptoms. While factor analyses can inform about dimensions of an underlying construct across a given population, cluster analyses help to better understand heterogeneity within a given population by identifying subgroups [18] and therefore allows a better patient typology of the clinical reality. Clustering algorithms have been employed to explore sub-structures of various psychopathological phenomena, e.g., for subtypes of depression [19] or cognitive subtypes in SZ [20] as well as transdiagnostic clustering approaches, focusing on cognition [21], psychotic symptoms [22] or other symptom domains and risk factors [23]. Although FTD is a prominent syndrome not only in SSD but also in BD and MDD, cluster analytic approaches to disentangle FTD symptom and severity subtypes across these disorders are lacking.

There are to our knowledge only two studies that have investigated FTD subtype clustering, restricted diagnostically to recent onset psychosis patients. By applying three different clustering algorithms (k-means, hierarchical, partitioning around medoids), the study by Oeztuerk et al. identified two subgroups distinguished by high vs. low FTD in recent onset psychosis and associated the former with lower neurocognitive performance, social and occupational functioning but not with global disease severity [24]. Subsequently, these two subtypes were predicted using GMV and functional MRI measures by Buciuman et al. The GMV and functional MRI measures of the salience, dorsal attention, visual, and ventral attention networks classified the two FTD subtypes with a combined multimodal balanced accuracy of 77% [25].

The vast majority of studies on the GMV correlates of FTD were done in SZ, however with heterogeneous findings. Among the most consistent results on the neuroanatomical correlates of FTD are GMV reductions in the bilateral superior temporal (STG) and inferior frontal gyri (IFG), partly corresponding to Wernicke’s and Broca’s language areas [3, 26–29]. This points to the assumption that structural aberrations of areas associated with language production and processing contribute to FTD. From a functional perspective, including both task-based and resting-state analyses, consistent activation changes have been observed in the left STG and in the posterior, ventral, and dorsal regions of the middle temporal gyrus (MTG) [30, 31].

From a more dimensional perspective, only few studies investigated associations of GMV and FTD across diagnoses. Hereof, a disorganization dimension was negatively correlated with the left middle occipital/angular gyrus, while emptiness (i.e., negative FTD) was negatively correlated with the left hippocampus/thalamus [7]. Moreover, we expanded this approach by investigating brain networks (i.e., GMV and white matter connections) being associated with these FTD dimensions across diagnoses [32]. Network-based analyses yielded subnetworks that predominantly comprised brain regions implicated in speech across both hemispheres [7, 32]. In contrast to these previous factor analytic studies from our group identifying dimensions (i.e., latent factors of FTD), the present study aims to elucidate subtypes of FTD by clustering individual patients based on FTD psychopathology into transdiagnostic groups.

Looking at other morphometric MRI measures such as cortical thickness, few studies are available investigating FTD in SZ and none across diagnoses. Using a network-based approach, Palaniyappan et al. (2020) reported the presence of positive FTD in SZ to be correlated with reduced cortical thickness in the language network located in the superior temporal cortex, and also related positive FTD severity with cortical thickness in the fronto-parietal network [33]. A comprehensive understanding of FTD requires the integration of both volume- and surface-based morphometric analyses. GMV reflects macroscopic structural changes associated with neurodevelopmental or adulthood processes, while surface-based metrics such as cortical thickness, sulcal depth, and gyrification capture finer details of cortical morphology and folding [17, 18, 34–36], usually linked to early (genetic and fetal) neurodevelopment. By combining these complementary methods, our study offers novel insights into its neuroanatomical basis.

Despite the presence of FTD in MDD, BD and SZ [6], there are – to the best of our knowledge – no studies available on transdiagnostic FTD clustering across different illness stages and none on their neuro-structural correlates, particularly not in a large patient sample. The present study’s aims were therefore (i) to identify FTD clusters (i.e., subtypes) in a large transdiagnostic sample (MDD, BD, SSD) using a model-based clustering algorithm; (ii) to characterize the FTD clusters with regard to psychopathology (other than FTD) and neurocognitive performance; and (iii) to explore associations between FTD clusters and GMV as well as cortical surface measures using MRI morphometric methods. For (i) it was expected that the sample would sub-divide into at least two clusters based on FTD symptomatology ranging from low to severe FTD. These clusters were (ii) hypothesized to differ in neuropsychological tests that have previously been shown to correlate with the presence of FTD in SZ, i.e., attention, executive function, working memory and verbal fluency. Finally, (iii) structural correlations in brain regions previously associated with negative and positive FTD in SZ patients, in particular in the extended language network, were expected across MDD, BD, and SSD diagnoses.

## Material and methods

### Participants

For the present analyses, participants from MACS cohort of the FOR2107 consortium, a bi-central study on the neurobiology of affective and psychotic disorders [37] were included. The sample comprised a broad spectrum of acutely ill to remitted patients from the Departments of Psychiatry and Psychotherapy, University Hospital of Marburg, and the Institute for Translational Psychiatry in Münster, both Germany as well as local participating hospitals and outpatient clinics (for details see [37]). All procedures were approved by the local Ethics Committees according to the Declaration of Helsinki and written informed consent was obtained from each subject. Patients with verbal IQ < 80, history of head trauma or unconsciousness, current substance dependence, current intake of Benzodiazepines, and neurological illness were excluded. Subsequent to a quality check of the T1-weighted images and the remove of incomplete data, 1 032 patients (aged 18–65) who met DSM-IV-TR criteria (SCID-I diagnosed) for MDD (*n* = 800, f = 529/m = 271), BD (*n* = 125, f = 66/m = 59), SSD (*n* = 107, SZ (*n* = 67, f = 30/m = 37) and SZA (*n* = 40, f = 22/m = 18)) (see Table 1) were analyzed.

**Table 1:** Sample characteristics (N = 1 032).

	MDD (*n* = 800)	BD (*n* = 125)	SSD (SZ/SZA) (*n* = 107)	Group comparison (F-value in brackets)
Age	36.55 (13.15)	41.04 (11.77)	38.07 (11.7)	*p* < 0.001^a^ (8.23)
Sex	271m / 529f	59m / 66f	55m / 52f	*p* < 0.001
Years of education	13.22 (2.71)	14.08 (2.77)	12.46 (2.71)	*p* < 0.001^b^ (9.91)
TIV	1560.46 (152.98)	1580.42 (146.19)	1580.23 (188.16)	*p* = 0.255 (1.37)
HAM-D sum	8.35 (6.38)	6.96 (5.9)	6.69 (5.79)	*p* < 0.01^c^ (4.56)
YRMS sum	1.43 (2.1)	3.9 (5.99)	2.41 (4.73)	*p* < 0.001^d^ (18.21)
SAPS sum	0.62 (2.01)	2.39 (4.36)	8.93 (11.48)	*p* < 0.001^e^ (156.35)
SAPS positive formal thought disorder	0.32 (1.3)	1.74 (3.25)	2.79 (3.99)	*p* < 0.001^e^ (77.28)
SAPS hallucinations	0.08 (0.5)	0.17 (0.79)	1.88 (4.13)	*p* < 0.001^f^ (75.99)
SAPS delusions	0.16 (0.87)	0.3 (0.85)	3.94 (5.99)	*p* < 0.001^f^ (154.86)
SANS sum	7.42 (8.67)	5.56 (7.13)	12.81 (11.54)	*p* < 0.001^f^ (14.69)
SANS alogia (negative formal thought disorder)	0.49 (1.3)	0.61 (1.32)	1.75 (2.6)	*p* < 0.001^f^ (33.1)
SANS anhedonia	2.9 (3.5)	1.94 (3.05)	2.78 (3.47)	*p* < 0.001^g^ (4.24)

Mean (standard deviation), SANS (Scale for the Assessment of Negative Symptoms), SAPS (Scale for the Assessment of Positive Symptoms), YMRS (Young Mania rating scale, HAM-D (Hamilton rating scale for Depression), TIV (Total intracranial volume).

^a^MDD < BD.

^b^MDD, SSD < BD.

^c^BD, SSD < MDD.

^d^MDD < BD, SSD; SSD < BD.

^e^MDD < BD, SSD; BD < SSD.

^f^SSD > BD, MDD.

^g^MDD > BD.

### Clinical and neuropsychological assessment

Psychopathology was assessed during a semi-structured clinical interview, incorporating SCID-I and various psychopathology scales. All interviewers were trained with the evaluation of the scales used. Positive and negative symptoms were assessed with the Scale for the Assessment of Positive Symptoms (SAPS) [15] and the Scale for the Assessment of Negative Symptoms (SANS) [14]. For the evaluation of manic and depressive symptoms, the Young Mania Rating Scale (YRMS) [38] and the Hamilton Rating Scale for Depression (HAM-D) [39] were employed, respectively. For the present analysis, the single items of the SANS alogia subscale and SAPS positive formal thought disorder subscale were used, as well as two items of the YMRS and one item of the HAM-D describing an impairment in the form of thought and language.

In addition, performance in several neurocognitive domains was assessed using a broad neuropsychological test battery. Verbal episodic memory was assessed with the German version of the California verbal learn- and memory retention test [40] including the sum of correct remembered words. Verbal working memory was tested using forward and reverse letter-number-spans [41] and visuospatial memory was assessed with a block span task [42]. Executive functions were evaluated using the Trail-Making-Test (TMT version A and B) [43]. During the D2-test for attention [44], participants were asked to cross out a target letter (“d”) among similar distractors, providing a measure of sustained and selective attention as well as accuracy. For the assessment of verbal fluency, the Regensburger verbal fluency test (RWT) [45] was employed, providing a measure of lexical (“name words starting with p”) and semantic (“name animals”) verbal fluency.

### Identification of formal thought disorder clusters (subtypes) using latent profile analysis

Latent profile analysis is a statistical technique for classification of individuals of a given population into homogenous latent clusters or subtypes based on a certain set of observed categorical (LCA) or continuous (LPA) variables, also referred to as indicators. As a model-based cluster analytic approach, it is a case of finite mixture modelling and provides more flexibility than traditional cluster algorithms, since it is based on an explicit model of the data [46]. It follows the assumption that the data is generated from a mixture of underlying probability distributions, where each component can be interpreted as a cluster that is more homogenous in the distribution of variable means, variances and covariances [47]. Subjects are assigned to these unobserved (latent) classes based on their probability of belonging to each class given the pattern of scores across indicator variables [48].

Here, latent profile analyses were performed using the R package mclust (version 5.4.7) [49], designed for model-based clustering, classification and density estimation based on finite Gaussian mixture modelling using the Expectation Maximization algorithm, as well as the R package tidyLPA (version 1.0.8) [50] which provides an interface to mclust functions for performing latent profile analysis. As indicators we used z-standardized items of the SANS, SAPS, YMRS, and HAM-D psychopathology scales assessing impairment of language and thought. Supplement 1 lists all latent profile indicators.

The decision upon the number of latent classes in a model and the evaluation of criteria in this process remains a matter of debate. Therefore, we used several criteria. These included the Bayesian Information Criterion (BIC) [51], the bootstrapped likelihood ratio test (BLRT, compares the improvement in model fit between a k–1 class model and k class model) [46], entropy (measure of classification uncertainty that should be close to 1) [52], the minimum of the average latent class probabilities for most likely class membership, also reflecting classification certainty [53], and the minimum class size (classes should not contain less than 5% of the sample, although it might be preferable to include smaller classes if they are clearly interpretable). All in all, while a number of fit indices and diagnostic criteria should be considered in order to make an objective decision on the number of classes, theoretical assumptions and interpretability of the model should also be taken into account during the process of model selection [54]. Therefore, we tested models including 2 to 5 classes and compared these models with regard to the above criteria. After final model selection, subjects were assigned to classes based on estimated posterior class membership probabilities and in a next step the resulting classes were entered into subsequent analyses with the aim to validate and characterize FTD clusters regarding clinical features and neurocognitive performance. Therefore, we used analyses of variance (ANOVA) and Tukey post-hoc tests in R to investigate differences between the identified clusters with regard to FTD symptoms, general psychopathology, and neuropsychological performance. In case assumptions for parametric testing were not met, a non-parametric approach was used. Correction for multiple testing was performed using the Benjamini-Hochberg approach [55].

As the DSM-IV-TR diagnostic groups were unequally distributed, we wanted to rule out potential confounding effects of formal diagnosis. Therefore, we re-ran clustering analysis in an age- and sex-matched sample with an equal diagnosis distribution (each *n* = 107 of MDD, BD, SSD, total N = 321) (see supplemental eTable 6). Matching was performed using the “MatchIt” package [56] in R [57]. In addition, FTD cluster analysis was applied in each diagnostic group separately.

### MRI acquisition and preprocessing

Details of MRI data acquisition and all preprocessing steps can be found in supplement 3 and 4. For the present study, we investigated the following surface measures as derived by CAT12: cortical thickness, sulcal depth, cortical complexity, and gyrification.

### Voxel and surface based morphometry statistical analyses

Morphometry analyses (GMV, cortical thickness, sulcus depth, gyrification, cortical complexity) were performed using the CAT12 toolbox as implemented in SPM12. Smoothed GM images were entered into a full factorial model (one-way ANOVA) with the groups obtained via the LPA included as one factor with four levels. As covariates of no interest, age, sex, total intracranial brain volume (TIV), and two dummy coded variables accounting for site and a body-coil exchange, as recommended by Vogelbacher et al. [58], were included in the analyses. To account for medication, we added the Medication Load Index (MedIndex) as additional covariate accounting type and amount of current medication adherence [59]. As surface values usually do not depend on TIV, it was not included as a covariate in SBM analyses (see CAT12 manual).

For second level analyses, an absolute threshold masking with a threshold value of 0.1 was applied. In order to detect differences in GMV and surface measures between latent clusters, voxel-wise parametric t-tests were calculated between each group obtained via the LPA. An initial cluster-forming threshold of *p* < 0.0001 was applied and results were corrected for multiple comparisons controlling family-wise error (FWE) at *p* < 0.05 cluster-level.

To investigate whether transdiagnostic brain correlates of FTD were driven by DSM-IV-TR diagnostic categories, we performed post-hoc interaction analyses using an ANCOVA design in R with the clinical DSM diagnosis and cluster membership as factors and significant clusters from VBM and SBM as dependent variables. Therefore, eigenvariate values approximating mean GMV and vertex values for surface measures of significant clusters were extracted. Due to the unbalanced distribution of DSM-IV-TR categories, we used whole brain clusters of the total sample and tested them as regions of interest (ROIs) in an equally distributed sample matched for age and sex. Using the batch mode, the search space was restricted for each significant cluster from the total sample beforehand. Same covariates as for whole brain analyses in the total sample were accounted for, using a threshold of *p* < 0.05, uncorrected with *k* ≥ 5.

## Results

### Modell comparison and selection

For the LPA, two different statistical models with different covariance structures were estimated. One restricted the variance to be equal across clusters and covariance to be zero, i.e., conditional independence of the indicator variables. For the other model type, covariance was additionally estimated and constrained to be equal across clusters. The latter showed an overall better fit than model 1 (covariance fixed to 0) for <5 clusters. For >5 clusters, model 1 showed a notable decrease in BIC and boundary estimates equaling 0 and 1, which is an indicator for convergence at a local instead of a global maximum [60].

**Fig. 1:**
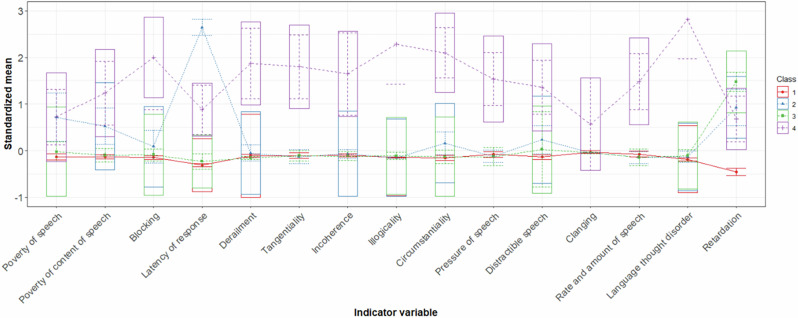
Four-cluster item profiles for FTD symptoms. Note: Estimated standardized sample means of SANS, SAPS, YMRS and HAM-D items used as indicator variables in the LPA across clusters. Boxes encompass +/− 1SD. Cluster 1 (*minimal* FTD), cluster 2 (*poverty*), cluster 3 (*inhibition*), cluster 4 (*severe* FTD).

The 4-cluster solution had a lower BIC, AIC, and a significant BLRT (*p* < 0.01), relative to the 3-class solution. With including additional clusters (5-cluster solution), AIC and BIC increased and entropy and minimum latent class posterior probability were lower than in the 4-cluster model (see eTable 1a for detailed fit indices). Therefore, the 4-cluster solution is presented here (Fig. 1). Cluster membership was extracted based on the estimated posterior latent class probability indicating most likely class membership for each subject (see eTable 1b and c).

**Fig. 2:**
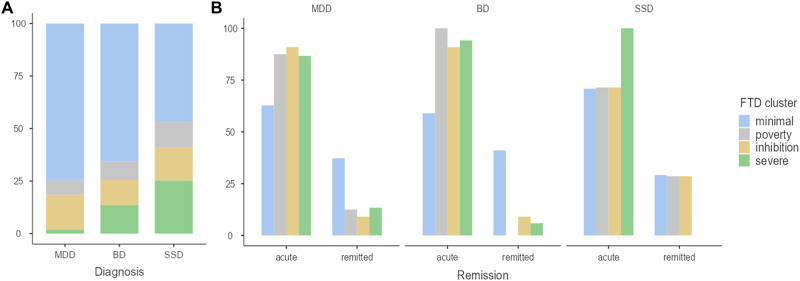
Distribution of diagnosis and clinical status across identified FTD clusters. **Note:** **A** Relative distribution of diagnoses. Diagnoses were distributed across all 4 clusters with MDD patients constituting the largest proportion of every cluster except for cluster 4 (*severe* FTD), where SSD patients outnumbered all other patient groups, while MDD patients composed the smallest percentage. **B** Acute/remitted patients within latent clusters.

In the 4-cluster solution, cluster 1 (*minimal FTD*) was the largest comprising 729 subjects (70.6%), followed by cluster 3 (*inhibition*) including 164 subjects (15.9%), and cluster 2 (*poverty*) constituted of 80 subjects (7.8%) of the sample. The smallest group was cluster 4 (*severe FTD*) with 59 subjects (5.7%). Supplemental eTable 1 summarizes the cluster sizes and estimated posterior latent class probability for each cluster. Figure 2a, b show the distribution of DSM-IV-TR diagnostic categories and remission status across clusters.

We re-ran our analyses in an age- and sex-matched subsample with equal representation across diagnostic groups and conducted separate analyses within each diagnostic group (MDD, BD, SSD). In all cases, the 4-cluster solution consistently emerged as the best fit, demonstrating the robustness of our transdiagnostic clustering model (see supplemental eTables 6–13 and eFigs. 3–6). Remarkably, 90% of participants in the matched subsample were assigned to the same cluster as in the full sample, underscoring the stability of the clustering solution across different sample compositions. In the matched subsample, cluster distribution closely mirrored that of the full sample: *minimal FTD* remained the largest, including 202 patients (62.9%), followed by *severe FTD* with 48 participants (15.0%), *poverty* with 41 participants (12.8%), and *inhibition* with 30 patients (9.3%). Consistent with our main findings, DSM-IV-TR diagnostic categories were represented across all four clusters in the matched sample.

### Formal thought disorder psychopathological characteristics

Clusters were compared with respect to average ratings across all indicator variables using ANOVA and Tukey post-hoc tests. Clusters differed significantly across all indicator variables used in the LPA (Fig. 1 for a visualization of the estimated latent FTD profiles, eFig. 1 and eTable 2 for unstandardized means and results of post-hoc tests). To summarize, *minimal* FTD showed no or only subclinical FTD symptomatology, whereas *poverty* was characterized by moderate FTD symptoms manifesting as a quantitative deficit of produced speech with especially increased latency of response but also signs of circumstantiality. Subjects of *inhibition* seemed to exhibit inhibited speech and thought process but at most a mild deficit in the amount of speech produced. *Severe* FTD reflected the most severe symptoms with a tendency towards but not exclusively positive FTD symptoms, manifesting as an overall increase of speech production with disorganized patterns of speech marked by circumstantial and disjointed utterances that convey little substantial information (Fig. 1). FTD psychopathological characteristics of the 4-cluster solution in the age- and sex-matched subsample with same n per diagnosis can be found in supplemental eTable 5.

### Clinical and demographic characteristics

Supplemental eTable 3 shows demographic and clinical characteristics of the identified clusters. Identified clusters differed significantly across all psychopathology rating scales and subscales included. In summary, *minimal FTD* showed less overall negative and positive symptoms as well as depressive symptoms compared to all other clusters. P*overty* was found to exhibit prominent negative symptoms compared to *minimal* FTD and *inhibition* and additionally exhibited a significantly higher average in hallucinations. Individuals of *inhibition* were found to have highest ratings in anhedonia but showed significantly lower ratings than *poverty* and *severe* FTD in all remaining subscales referring to positive and negative symptoms. Clinical and demographic characteristics of the 4-cluster solution in the age- and sex-matched subsample with same n per diagnosis can be found in supplemental eTable 11.

### Neurocognitive characteristics

Clusters were further compared with respect to performance in neuropsychological domains that have previously been correlated with FTD severity in SZ. Subjects in *minimal* FTD performed significantly better than all other cluster in the domains of phonemic verbal fluency and attention. Clusters 2 and 4 showed significantly lower scores than clusters 1 and 3 in terms of executive function, working memory, semantic verbal fluency, and episodic verbal memory (supplemental eFig. 2). Overall, those clusters that were characterized by *poverty* and *severe* FTD could also be distinguished by poor neurocognitive functioning across all inspected domains (eTable 4). Neurocognitive characteristics of the 4-cluster solution in the age- and sex-matched subsample with same n per diagnosis can be found in supplemental eTable 12.

### Association of formal thought disorder clusters with GMV and sulcal depth

**Fig. 3:**
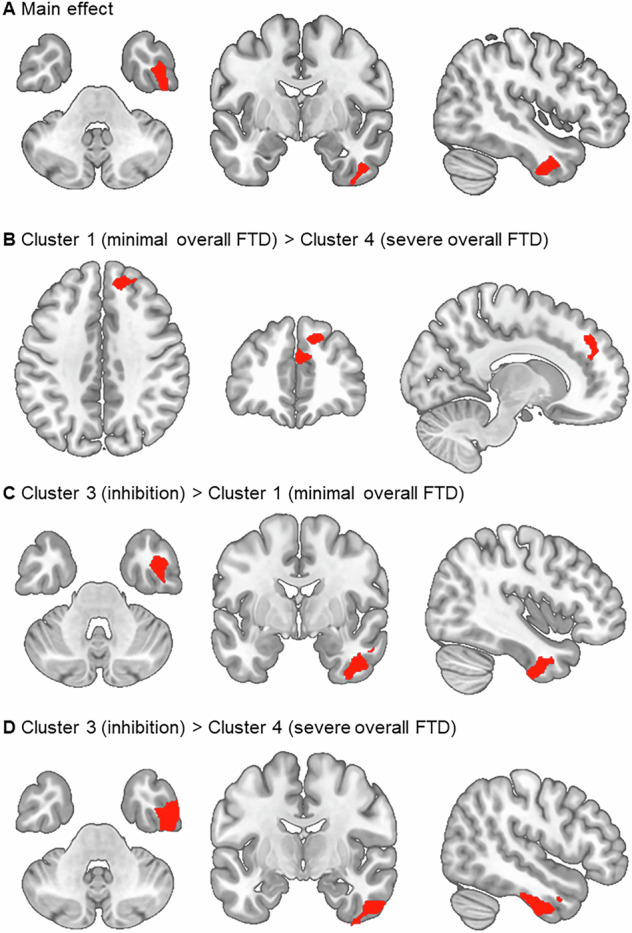
Associations of FTD clusters with gray matter volume. Note: Significant GMV clusters at *p* < 0.05 cluster-level FWE-corrected (initial cluster defining threshold of *p* < 0.0001). **A** Main effect of FTD cluster; **B** cluster 1 (*minimal overall FTD*) > cluster 4 (*severe overall FTD*); **C** cluster 3 (*inhibition*) > cluster 1 (*minimal overall FTD*); **D** cluster 3 (*inhibition*) > cluster 4 (*severe overall FTD*); FTD formal thought disorder, FWE Family-Wise-Error.

**Fig. 4:**
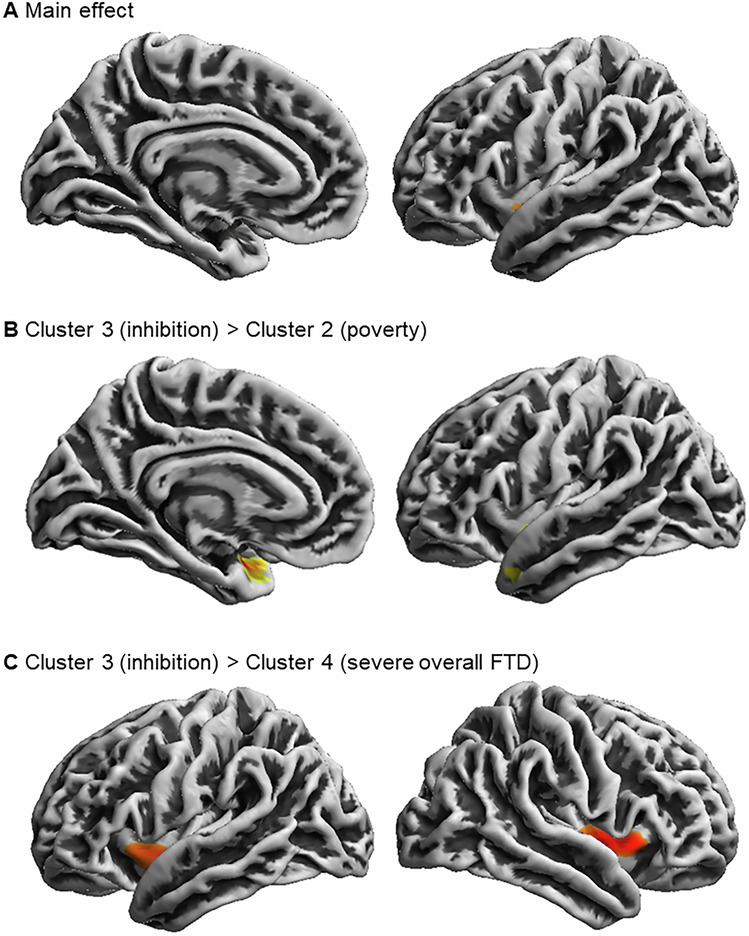
Associations of FTD clusters with sulcal depth. Note: Significant sulcal depth clusters at *p* < 0.05 cluster-level FWE-corrected (initial cluster defining threshold of *p* < 0.0001). **A** Main effect of FTD cluster; **B** cluster 3 (*inhibition*) > cluster 2 (*poverty*); **C** cluster 3 (*inhibition*) > cluster 4 (*severe overall FTD*); FTD formal thought disorder, FWE Family-Wise-Error.

Morphometry analyses revealed significant differences between the identified FTD clusters in GMV and sulcal depth (Figs. 3 and 4). We identified one FTD cluster main effect cluster for GMV (*k* = 844, x/y/z = 45/−6/−38, *p* = 0.012 FWE cluster-level corrected) comprising the right inferior and middle temporal gyri and the right fusiform gyrus. For sulcal depth, we identified a significant cluster comprising the left insula (*k* = 110, x/y/z = −37/1/−21, *p* = 0.045 FWE cluster-level corrected). Post-hoc parametric t-tests revealed GMV and sulcal depth clusters that differed significantly between the *poverty* and *inhibition* FTD cluster and clusters with *minimal* and *severe* FTD, respectively. Investigations of cortical thickness, gyrification, and complexity did not result in significant effects. Table 2 lists all significant effects.

**Table 2:** Differences between identified FTD clusters in GMV and sulcal depth.

	H	x	y	z	*t*	*k*	*p* FWE cluster-level	*p* inter-action analyses	*Partial. η²*
**GMV**									
cluster 1 (*minimal overall FTD*) > cluster 4 (*severe overall FTD*)									
55.5% superior frontal gyrus 44.5% medial frontal gyrus	R	12	42	38	3.99	800	0.048	0.404	0.022
cluster 3 (*inhibition*) > cluster 1 (*minimal overall FTD*)									
50.8% inferior temporal gyrus 19.5% fusiform gyrus 18.2% middle temporal gyrus	R	42	−3	−34	4.93	926	0.029	0.060	0.027
cluster 3 (*inhibition*) > cluster 4 (*severe overall FTD*)									
85.6% inferior temporal gyrus 11.5% middle temporal gyrus 2.9% fusiform gyrus	R	48	−9	−36	4.45	2404	**<0.0001**	0.063	0.020
**Sulcal depth**									
cluster 3 (*inhibition*) > cluster 2 (*poverty*)									
45% insula 37% superior temporal sulcus 16% temporal pole	L	−36	1	−22	3.97	238	**0.004**	0.145	0.019
cluster 3 (*inhibition*) > cluster 4 (*severe overall FTD*)									
100% insula	R	40	5	−2	3.57	287	**0.001**	0.778	0.009
100% insula	L	−39	2	−7	3.31	188	**0.001**	0.667	0.005

*Note*: Interaction analyses refer to the post-hoc investigation of clinical DSM diagnoses on brain structural differences between FTD clusters. Therefore, an ANCOVA design in R with the clinical DSM diagnosis and cluster membership as factors and significant clusters from VBM and SBM as dependent variables were used. *R* right, *L* left, *H* hemisphere; *FWE* Family-Wise-Error; *k* cluster extend; bold letters indicate significance after correction for multiple testing (Benjamini Hochberg).

To further confirm the stability of our neuroanatomical findings, we analyzed brain structural differences across FTD clusters within the age- and sex-matched subsample, using the significant clusters identified in the total sample as ROIs. All significant neuroanatomical clusters from the total sample were successfully replicated in the matched subsample (see supplemental eTable 13).

## Discussion

The aim of the present study was to identify latent patient clusters (i.e., psychopathological subtypes) based on FTD psychopathology in a large transdiagnostic sample of patients with MDD, BD, and SSD, and to investigate gray matter as well as surface brain structure associated with the identified FTD clusters. There were four FTD clusters, i.e., *minimal* and *severe* FTD, while the other two manifested as a low amount of produced speech (*poverty*) and inhibited speech and thought process (*inhibition*). Identified FTD clusters differed in neurocognitive domain test performance as well as GMV and sulcal depth patterns. sMRI analyses revealed differences between the *inhibition* cluster compared to both *minimal* and *severe* FTD in the right inferior temporal lobe. *Minimal* and *severe* FTD differed in the GMV of the right superior frontal lobe. Sulcal depth was reduced around the left insula (*inhibition* vs. *poverty*) as well as bilaterally (*inhibition* vs. *severe* FTD). Psychopathological clusters and their neural correlates were present across diagnosis and stable when correcting for current psychotropic medication. Additionally, robustness analyses confirmed that the four-cluster solution remained valid in an age- and sex-matched subsample and within each diagnosis independently. Furthermore, the neuroanatomical findings were replicated in the matched subsample, demonstrating that the observed structural differences across FTD clusters were consistent and not influenced by sample imbalances.

Based on our findings, several new insights emerge. First, identified FTD clusters were not distinguished with regard to positive and negative FTD, but rather differed in their severity of all FTD symptoms while showing a tendency towards one symptom (e.g., poverty of speech). In contrast to factor analytic studies which aggregate symptoms, we could demonstrate that when clustering individual patients, pFTD and nFTD occur concomitantly in the same patient. Interestingly, Oeztuerk et al. [24] also identified low vs. high FTD clusters in recent-onset psychosis. In their study, high FTD was characterized by conceptual disorganization, poverty of speech and poverty of content of speech, and increased response latency, similarly to our results of the *severe* cluster [24]. We further identified one distinctly negative FTD patient cluster who exhibited mainly *poverty*, in line with factor analytic studies in SZ [11] as well as one *inhibition* patient cluster. Importantly, diagnoses were distributed across the identified latent clusters such that every DSM-IV-TR diagnosis was present in each FTD cluster, indicating that FTD is not an exclusive feature of SSD or bipolar mania [2, 7]. *Severe* FTD, collapsing acute and remitted patients, comprised a higher percentage of BD and SSD vs. MDD subjects suggesting that patterns of severe disorganized speech are more prevalent in these disorders [8] which is unsurprising because this is part of the DSM-IV/V diagnostic classification. Yet strikingly, both, the *severe* and the *minimal* clusters are distributed almost evenly across the three diagnostic categories in the acute patients (Fig. 2b). Additionally, results showed that each FTD cluster was present even in remission across diagnoses, underscoring the significance of FTD across various illness stages.

Second, regarding associations of patient clusters with neuropsychological test domain performance, *poverty* and *severe* FTD were characterized by poor performance in lexical and semantic verbal fluency, attention, executive functioning, working memory and verbal episodic working memory. This supports the notion of FTD being related to or being -in part- a result of impaired neurocognitive functioning (e.g., dysexecutive functioning, impaired semantic access) [3, 61–63], not only in SSD patients but also in affective disorders (i.e., transdiagnostic samples) [64, 65].

Third, as hypothesized, the most prominent gray matter structural aberrations were found in the *severe* FTD symptom cluster: In comparison to the *minimal* FTD patients, they showed reduced GMV in the right superior/medial frontal and to the *inhibition* cluster in the right inferior/middle temporal gyri. Volume reductions in both the temporal and frontal lobes have been highly consistent findings in association with FTD in SZ [3, 26]. Not only for objective FTD ratings but also when investigating subjective aspects of FTD for example using the TALD scale [29]. While the MTG is part of the semantic system [66] and constitutes one of the core structures being implicated in the ventral stream of the language network [32] mapping phonological representations onto lexical conceptual representations [67], the right inferior temporal gyrus (ITG) has been correlated with the articulation rate in SZ during speech production tasks [68]. While the present study focused on structural MRI correlates of FTD, functional neuroimaging studies have demonstrated altered activity patterns in almost similar brain regions as reported here [3, 26, 28, 30, 31, 61, 69, 70]. For example, an activation likelihood estimation meta-analysis on FTD highlighted the role of the dorsal and ventral MTG in semantic processing of language related information [31].

Regarding cortical surface, patients’ brains in the *poverty* and *severe* FTD clusters showed sulcal flattening around the left insula, STS, temporal pole, and bilateral insula when compared to *inhibition*. This is in line with previous studies finding the bilateral GMV of the insula [29, 71] and cerebral blood flow in the right insula (along with MTG) [29, 72] to correlate with negative FTD. Sulcal changes have been associated with early neurodevelopmental impacts [73, 74], and in particular insular alterations with negative symptoms and poor psycho-social functioning in SZ patients [75, 76]. We could now extend and specify these findings, as our analyses revealed sulcal flattening in these clusters to be associated with negative FTD symptomatology across MDD, BD, and SSD. More generally our structural findings indicate that particular FTD syndromes may arise from distinct brain structural changes. This aligns well with our previous findings on structural network connectivity being associated with transdiagnostic FTD dimensions [32]. However, while our findings indicate specific differences in brain structure across FTD clusters, the cause remains speculative. Future longitudinal studies could clarify these relationships and help determine directionality.

Finally, differences in brain structure between FTD clusters were present across clinical DSM-IV-TR diagnoses and were independent of current medication. Brain regions that have been found to display reduced volume in SZ, were associated with FTD clusters across diagnoses, indicating that FTD related structural aberrations do not confine to psychotic disorders. This finding aligns with studies indicating a large (biological) overlap across affective and psychotic disorders including shared genetics [77], environmental risk factors [78], brain structural changes [7, 79–84], and psychopathology [23, 85].

### Future directions

Future research should investigate the stability of FTD clusters over time with the examination of sociodemographic factors as potential modulators. Longitudinal studies within and across specific diagnostic groups could provide valuable insights into how FTD clusters evolve with illness progression or in response to treatment. While FTD has been closely linked to illness progression in SZ, we suggest that its significance may (similarly) extend to other conditions, such as MDD.

### Limitations

The present study has some limitations. First, FTD was not assessed with a specific FTD scale but with 15 items devoted to language and thought-related symptoms from general psychopathology scales. Including items from specific FTD scales (such as the TALD scale [6]) in cluster analyses might lead to the identification of somewhat different FTD clusters, although the SAPS/SANS used in our study correlate highly with TLC and TALD. However, we have used widely recognized scales enhancing the generalizability and practical relevance of our findings. Second, since there is a lack of consensus about the criteria by which the correct model should be selected in LPA, the decision on the number of extracted clusters might be to a small extent subjective. While several criteria were evaluated in the current study, model selection was also guided by interpretability of FTD clusters. Third, aberrations in gray matter brain structure between FTD clusters resulted from group comparisons without healthy participants, because of the small variance in FTD present in the latter group [3, 6]. Nevertheless, this approach is most suitable for the exploration of differences between FTD clusters across mental disorders. Fourth, the cross-sectional design limits the ability to draw conclusions about causality or directionality between neuroanatomical differences and FTD symptomatology. Fifth, the majority of patients included to the present study were diagnosed with MDD. Nevertheless, several robustness checks confirmed the transdiagnostic validity of FTD clusters.

## Conclusion

This study offers several strengths and new insights. First, data-driven approaches as employed in the present study provide valuable insight into the clinical co-occurrence of psychopathologies highlighting their nature as multidimensional constructs. By employing a transdiagnostic perspective, this work demonstrates how advanced clustering methods can reveal the heterogeneity within and across psychiatric disorders and therefore assist the establishment of symptom-specific typologies (i.e., syndromes and sub-syndromes), their neural correlates, and eventually treatments (which are mostly syndrome based in psychiatry). Second, combining data-driven approaches on psychopathology with the examination its of neuroanatomical correlates, our study contributes to understanding the neural basis of complex syndromes like FTD that transcends traditional diagnostic boundaries. Rather than aligning with any single diagnosis, our findings indicate that a network of structural brain changes is more closely associated with specific symptoms or syndromes, reinforcing the importance of a neurobiological transdiagnostic approach in future etiological research. Finally, we extended existing knowledge on the neural correlates of FTD by demonstrating that cortical surface alterations, in addition to volumetric changes, are implicated in thought and language impairment.

## Supplementary information

Supplement

## Data Availability

The raw data collected in this study is not openly accessible to protect participant consent and confidentiality. Nevertheless, the FOR2107 consortium is a highly valuable resource for the global research community and is, in principle, available to qualified scientific researchers affiliated with non-commercial research organizations worldwide. Researchers interested in accessing the data must submit a formal research proposal that clearly defines the specific research questions, methodology, and intended statistical analyses. Applications are reviewed by the study's principal investigators, please contact Professors Tilo Kircher and Udo Dannlowski for further information.

## Code availability

See supplementary materials for R code used in the LPA. Other codes and data used for the present analyses as well as generated outputs are available. Please contact the corresponding author (FS) for access.
