# Supplementary material for: Transdiagnostic types of formal thought disorder and their association with gray matter brain structure: a model-based cluster analytic approach
Source: Mol Psychiatry. 2025 Apr 11;30(9):4286–95. doi: 10.1038/s41380-025-03009-w (PMC12339403; doi:10.1038/s41380-025-03009-w)
Supplement: Supplementary file 1 — Supplement [file 41380_2025_3009_MOESM1_ESM.docx]

**Supplement**

[Supplement 1: Latent profile indicators 2](#_Toc183358538)

[Supplement 2: R Code used for LPA analyses 3](#_Toc183358539)

[Supplement 3: Preprocessing of MRI data sets 5](#_Toc183358540)

[eTable1a: Fit indices of the competing models (equal variance and covariance across clusters). 7](#_Toc183358541)

[eTable1b: Cluster size and latent cluster probabilities. 7](#_Toc183358542)

[eTable1c: Distribution of clinical diagnoses (DSM-IV-TR) across latent FTD clusters. 7](#_Toc183358543)

[eFigure1: Distribution of four-cluster item profiles for FTD symptoms. 8](#_Toc183358544)

[eFigure2: Neurocognitive profiles of FTD subtypes. 9](#_Toc183358545)

[eTable2: FTD psychopathological characteristics of latent clusters. 10](#_Toc183358546)

[eTable3: Demographic and clinical characteristics of latent clusters. 11](#_Toc183358547)

[eTable4: Neurocognitive characteristics of FTD clusters. 12](#_Toc183358548)

[eTable5: Sample characteristics of the age- and sex-matched subsample with same n per diagnosis (N=321). 13](#_Toc183358549)

[eTable6: Fit indices of the competing models (equal variance and covariance across clusters) in the age- and sex-matched subsample N=321. 14](#_Toc183358550)

[eTable7: FTD psychopathological characteristics of latent clusters in an age- and sex-matched sample with same n per DSM-IV-TR diagnoses (N=321). 14](#_Toc183358551)

[eFigure3: Four-cluster item profiles for FTD symptoms in the age- and sex-matched sample with same n per DSM-IV-TR diagnoses (N=321). 15](#_Toc183358552)

[eFigure4: Elbow plot for different cluster models in the age- and sex-matched sample with same n per DSM-IV-TR diagnoses (N=321). 16](#_Toc183358553)

[eFigure5: Distribution of four-cluster item profiles for FTD symptoms in the age- and sex-matched subsample with same n per diagnosis (N=321). 17](#_Toc183358554)

[eFigure6: Relative distribution of clinical diagnoses within latent FTD clusters in the age- and sex-matched subsample with same n per diagnosis (N=321). 18](#_Toc183358555)

[eTable8: Fit indices of the competing models (equal variance and covariance across clusters) in MDD (n=800). 18](#_Toc183358556)

[eTable9: Fit indices of the competing models (equal variance and covariance across clusters) in BD (n=125). 18](#_Toc183358557)

[eTable10: Fit indices of the competing models (equal variance and covariance across clusters) in SSD (n=107). 19](#_Toc183358558)

[eTable11: Demographic and clinical characteristics of latent FTD clusters in the age- and sex-matched subsample with same n per diagnosis (N=321). 19](#_Toc183358559)

[eTable12: Neurocognitive characteristics of FTD clusters in the age- and sex-matched subsample (N=321). 20](#_Toc183358560)

[eTable 13: Differences between identified clusters in GMV and sulcal depth in the age- and sex-matched subsample (N=321). 21](#_Toc183358561)

[References 22](#_Toc183358562)

# Supplement 1: Latent profile indicators

Z-standardized values of 15 symptoms assessing impairment of language and thought from the SAPS sub-scale for positive formal thought disorder, SANS sub-scale for alogia/paralogia, the YRMS and HAMD were included as indicator variables. Items referring to a positive symptom dimension of FTD included derailment (SAPS 26), tangentiality (SAPS 27), incoherence (SAPS 28), illogical thinking (SAPS 29), circumstantiality (SAPS 30), pressure of speech (SAPS 31), distractible speech (SAPS 32) and clanging (SAPS 33), as well as the rate and amount of speech (YRMS 6) and the rating of language-thought-disorder (YRMS 7). Items describing a negative symptom dimension of FTD were poverty of speech (SANS 8), poverty of content of speech (SANS 9), blocking (SANS 10), increased latency of response (SANS 11) and retardation (HAMD 16).

# Supplement 2: R Code used for LPA analyses

| R code used for latent profile analysis with tidyLPA and mclust and data visualization. |
| --- |
| rm(list = ls())  library(tidyLPA)  library(dplyr)  library(haven)  library(readxl)  library(tidyverse)  library(mclust)  library(ggplot2)  #set path  setwd("")  #load data  data <- read_sav("data_lpa.sav")  #estimate models with varying number of profiles and compare fit statistics of solutions  LPA <- data %>%  select(ZSANS8, ZSANS9, ZSANS10, ZSANS11, ZSAPS26, ZSAPS27, ZSAPS28, ZSAPS29, ZSAPS30, ZSAPS31, ZSAPS32, ZSAPS33, ZYMRS6, ZYMRS7, ZHAMD16) %>%  single_imputation() %>%  estimate_profiles(1:7,  variances = c("equal", "varying", "equal", "varying"),  covariances = c("zero", "zero", "equal", "varying"))  compare_solutions(LPA, statistics = c("AIC", "BIC", "SABIC", "Entropy", "prob_min", "prob_max", "n_min", "n_max", "BLRT_p"))  plot(LPA)  #estimate selected model (model 3: equal variance and covariance)  model3 <- data %>%  select(ZSANS8, ZSANS9, ZSANS10, ZSANS11, ZSAPS26, ZSAPS27, ZSAPS28, ZSAPS29, ZSAPS30, ZSAPS31, ZSAPS32, ZSAPS33, ZYMRS6, ZYMRS7, ZHAMD16)%>%  single_imputation() %>%  estimate_profiles(4,  variances = "equal",  covariances = "equal")  #extract and save estimated data  getdata <- get_data(model3)  getestimates <- get_estimates(model3)  getfit <- get_fit(model3)  write_sav(getdata, "getdata.sav")  write_sav(getestimates, "getestimates.sav")  write_sav(getfit, "getfit.sav")  #plot results  plot_profiles(model3, variables = NULL , ci = 0.95, sd = TRUE, add_line = TRUE, rawdata = TRUE, bw = FALSE, alpha_range = c(0,0.1))+ ylim(c(-1,10)) +  xlab("Indicator variable") +  ylab("Standardized mean") +  theme(axis.text = element_text(size = 16)) +  theme(axis.title = element_text(size = 16, face = "bold")) +  theme(axis.text.x = element_text(angle = 45, hjust = 1, size = 16))+  scale_x_discrete(limits = c("Poverty of speech", "Poverty of content of speech", "Blocking", "Latency of response", "Derailment", "Tangentiality", "Incoherence", "Illogicality", "Circumstantiality", "Pressure of speech", "Distractible speech", "Clanging", "Rate and amount of speech", "Language thought disorder","Retardation"))  # Create a list of variables and their corresponding labels  library(RColorBrewer)  library(patchwork)  variable_labels <- c(  "ZSANS8" = "Poverty of speech",  "ZSANS9" = "Poverty of content of speech",  "ZSANS10" = "Blocking",  "ZSANS11" = "Latency of response",  "ZSAPS26" = "Derailment",  "ZSAPS27" = "Tangentiality",  "ZSAPS28" = "Incoherence",  "ZSAPS29" = "Illogicality",  "ZSAPS30" = "Circumstantiality",  "ZSAPS31" = "Pressure of speech",  "ZSAPS32" = "Distractible speech",  "ZSAPS33" = "Clanging",  "ZYMRS6" = "Rate and amount of speech",  "ZYMRS7" = "Language thought disorder",  "ZHAMD16" = "Retardation")  # Iterate over each variable and create violin plots  plots <- lapply(names(variable_labels), function(var) {  ggplot(LPA, aes(x = factor(Class4_final), y = !!sym(var), fill = factor(Class4_final))) +  geom_violin(trim = FALSE, scale = "width", width = 0.7) +  geom_jitter(position = position_jitter(width = 0.2), color = "black", size = 0.5, alpha = 0.5) +  # Jittered raw data points  stat_summary(fun = mean, geom = "point", shape = 20, size = 3, color = "black") +  # Mean points  labs(x = "Class", y = variable_labels[var], fill = "Class") + # Renamed x-axis label and legend title + theme_minimal() + scale_y_continuous(limits = c(-1, 10), breaks = seq(-1, 10, by = 1)) +  # Adjust scaling and breaks on y-axis  ylim(-1, 10) + # Set y-axis limits  scale_fill_brewer(palette = "PuBuGn") + # Use PuBuGn color palette  # Remove legend  guides(fill = FALSE)  })  # Display the plots  plots  # Combine multiple plots into one without  combined_plot <- plots[[1]] + plots[[2]] + plots[[3]] + plots[[4]] + plots[[5]] + plots[[6]] +  plots[[7]] + plots[[8]] + plots[[9]] + plots[[10]] + plots[[11]] + plots[[12]] +  plots[[13]] + plots[[14]] + plots[[15]] +  plot_layout(ncol = 3, byrow = TRUE) # Adjust the layout to display plots in multiple rows  combined_plot |

# Supplement 3: MRI data acquisition

T1-weighted images were acquired using a 3 T MRI scanner (Marburg: Tim Trio, Siemens, Erlangen, Germany; Münster: Prisma, Siemens, Erlangen, Germany). In Marburg, a 12-channel head matrix Rx-coil, in Münster, a 20-channel head matrix Rx-coil was used. Following a quality assurance protocol, based on regular measurements of an MRI phantom [60], whole-brain T1-weighted scans were obtained using a fast gradient echo MP-RAGE sequence with a slice thickness of 1.0 mm, 176 (at Münster site: 192) sagittal orientated slices and FOV of 256 mm with the following parameters: Marburg: TR = 1.9 s, TE = 2.26 ms, TI = 900 ms, flip angle = 8°; Münster: TR = 2.13 s, TE = 2.28m s, TI = 900 ms, flip angle = 9°.

# Supplement 4: Preprocessing of MRI data sets

For all pre-processing steps, the default parameters as implemented in CAT12 (Computation Anatomy Toolbox for SPM, build 1184, Christian Gaser, Structural Brain Mapping Group, Jena University Hospital, Germany, http://dbm.neuro.uni-jena.de/cat/) were employed. In an initial voxel-based processing step, a spatial adaptive non-local means (SANLM) denoising filter was applied, followed by internal resampling to integrate low-resolution images and anisotropic spatial resolutions. After the correction of intensity inhomogeneity in T1-weighted images, resulting from inhomogeneities of the magnetic field, also referred to as bias correction [1], T1 weighted images were segmented into white matter (WM), grey matter (GM) and cerebrospinal fluid (CBF), using the standard unified segmentation [2]. In a refined voxel-based processing step, skull-stripping and regional parcellation of the brain into left and right hemisphere, cerebellum and subcortical areas were performed on the segmented data and possible local white matter hyperintensities were detected. Subsequently, a local intensity transformation of all tissue classes was conducted, followed by a final maximum a posteriori estimation of tissue segmentation [3]. In order to estimate the amount of each tissue type per voxel, a partial volume estimation [4] was employed. Finally, images were spatially normalized using DARTEL [5]. This involved registering all images to the same template by estimating a 12-parameter affine transformation [6].

For SBM analyses, images underwent an additional surface-based processing step. Cortical thickness estimation and surface reconstruction were obtained by applying a projection-based thickness method: First, white matter distance was estimated on the basis of tissue segmentation. Second, local maxima were projected onto gray matter voxels, employing a neighbouring relationship described by the WM distance [7]. In order to repair topological deficits, that might result from the generation of surface meshes, a topological correction method was applied, which modifies the uncorrected spherical maps of the brain and patches a low-pass filtered alternative reconstruction based on spherical harmonics into the surface reconstruction [8]. Finally, using spherical mapping with minimal distortions [9], the central surfaces were spatially registered to the FreeSurfer FsAverage template. Images were smoothed with a Gaussian kernel of 8 mm FWHM for GMV analyses and 20 mm for SBM analyses.

**Supplementary Figures and Tables**

# eTable1a: Fit indices of the competing models (equal variance and covariance across clusters).

| **Number of clusters** | **AIC** | **BIC** | **Entropy** | **prob. min.** | ***n* min.** | **BLRT p-value** |
| --- | --- | --- | --- | --- | --- | --- |
| 2 | 35638.8005 | 36300.6606 | .9913 | .9938 | .1502 | .0099 |
| 3 | 34692.2757 | 35428.2145 | .9940 | .9998 | .0756 | .0099 |
| **4** | **33794.0817** | **34604.1194** | **.9933** | **.9992** | **.0659** | **.0099** |
| 5 | 33954.3831 | 34838.5096 | .9688 | .9970 | .0601 | 1 |

# eTable1b: Cluster size and latent cluster probabilities.

| **Cluster** | **n (%) based on posterior probability** | **Cluster 1** | **Cluster 2** | **Cluster 3** | **Cluster 4** |
| --- | --- | --- | --- | --- | --- |
| 1 | 729 (70.6%) | .9957 | .0014 | .0028 | .0000 |
| 2 | 80 (7.8%) | .0007 | .9985 | .0008 | .0000 |
| 3 | 164 (15.9%) | .0945 | .0030 | .9025 | .0000 |
| 4 | 59 (5.7%) | .0000 | .0000 | .0000 | 1.0000 |

# eTable1c: Distribution of clinical diagnoses (DSM-IV-TR) across latent FTD clusters.

|  | | | | | | | | | | | | | |
| --- | --- | --- | --- | --- | --- | --- | --- | --- | --- | --- | --- | --- | --- |
|  | | | | **FTD Cluster** | | | | | | | |  | |
| **Diagnosis** | |  | | **minimal** | | **poverty** | | **inhibition** | | **severe** | | **Total** | |
| MDD |  | N |  | 597 |  | 56 |  | 132 |  | 15 |  | 800 |  |
|  | | % |  | 74.625 % |  | 7.000 % |  | 16.500 % |  | 1.875 % |  | 100.000 % |  |
| BD |  | N |  | 82 |  | 11 |  | 15 |  | 17 |  | 125 |  |
|  | | % |  | 65.600 % |  | 8.800 % |  | 12.000 % |  | 13.600 % |  | 100.000 % |  |
| SSD |  | N |  | 50 |  | 13 |  | 17 |  | 27 |  | 107 |  |
|  | | % |  | 46.729 % |  | 12.150 % |  | 15.888 % |  | 25.234 % |  | 100.000 % |  |
| Total sample |  | N |  | 729 |  | 80 |  | 164 |  | 59 |  | 1032 |  |
|  | | % |  | 70.640 % |  | 7.752 % |  | 15.891 % |  | 5.717 % |  | 100.000 % |  |
|  | | | | | | | | | | | | | |

# eFigure1: Distribution of four-cluster item profiles for FTD symptoms.


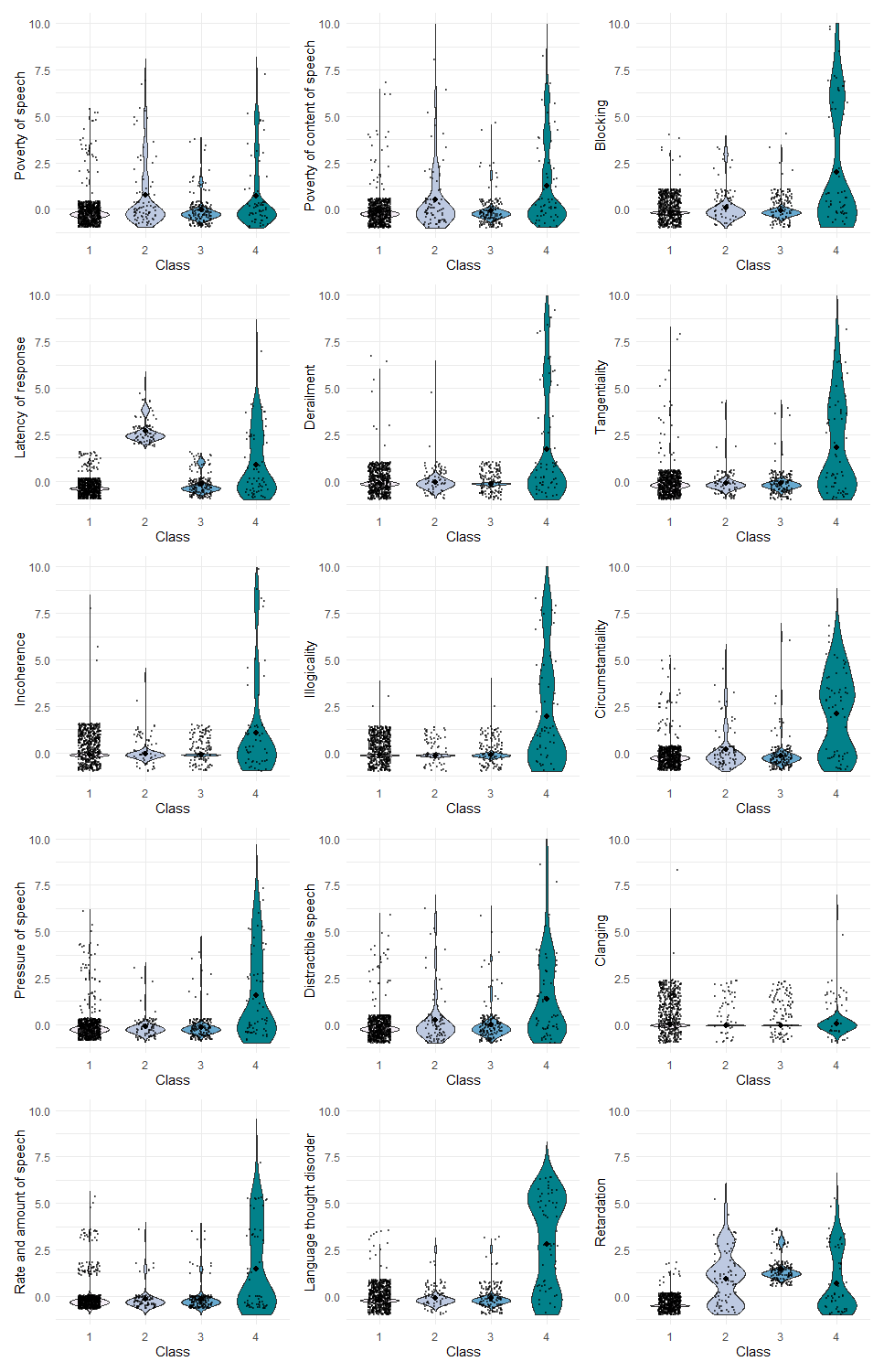


# eFigure2: Neurocognitive profiles of FTD subtypes.

*
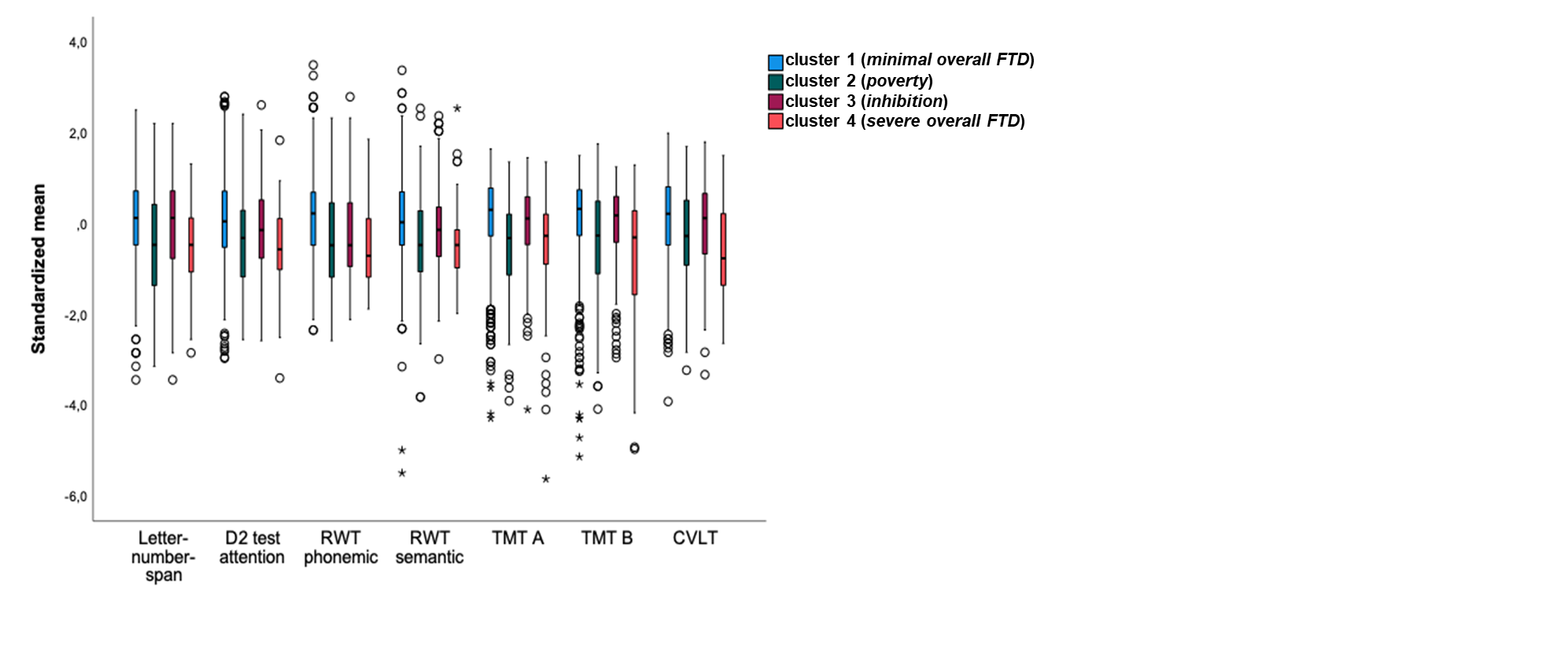
*

Note: Boxplots of standardized means within latent clusters across neuropsychological tests for working memory, attention, verbal fluency, executive function, and verbal episodic memory. Outliers are indicated by circles, extreme outliers by stars. RWT (Regensburger Wortflüssigkeitstest), TMT (Trail-Making-Test), CVLT (California Verbal Learning Test). Outliers could be identified particularly in the domain of executive function and verbal fluency.

# eTable2: FTD psychopathological characteristics of latent clusters.

|  | **Cluster 1 (minimal FTD)** | **Cluster 2**  **(poverty)** | **Cluster 3**  **(inhibition)** | **Cluster4**  **(severe FTD)** | **Group comparison  (F-value )** |
| --- | --- | --- | --- | --- | --- |
| Poverty of speech (SANS 8) | .09 (.43) | .59 (1.05) | .13 (.41) | .58 (1.07) | p<.001*** (30.04)^a^ |
| Poverty of content of speech (SANS 9) | .05 (3.18) | .38 (.8) | .07 (.31) | .71 (1.08) | p<.001*** (47.27)^a^ |
| Blocking (SANS 10) | .01 (.1) | .09 (.28) | .03 (.17) | .69 (1.02) | p<.001*** (114.93)^b^ |
| Latency of response (SANS 11) | .06 (.24) | 2.19 (.42) | .16 (.37) | .92 (1.22) | p<.001*** (783.7)^c^ |
| Derailment (SAPS 26) | .02 (.17) | .04 (.25) | .0 (.0) | .69 (1.1) | p<.001*** (89.57)^b^ |
| Tangentiality (SAPS 27) | .06 (.37) | .05 (.27) | .05 (.1) | 1.02 (1.23) | p<.001*** (86.56)^b^ |
| Incoherence (SAPS 28) | .01 (.09) | .01 (.11) | .0 (.0) | .42 (.86) | p<.001*** (68.94)^b^ |
| Illogicality (SAPS 29) | .0 (.05) | .0 (.0) | .01 (.08) | .63 (.87) | p<.001*** (150.08)^b^ |
| Circumstantiality (SAPS 30) | .08 (.4) | .28 (.66) | .09 (.43) | 1.46 (1.3) | p<.001*** (129.13)^d^ |
| Pressure of speech (SAPS 31) | .13 (.55) | .1 (.41) | .09 (.44) | 1.24 (1.59) | p<.001*** (59.0)^b^ |
| Distractible speech (SAPS 32) | .07 (.35) | .25 (.7) | .14 (.49) | .85 (1.16) | p<.001*** (48.81)^e^ |
| Clanging (SAPS 33) | .0 (.05) | .0 (.0) | .0 (.0) | .1 (.66) | p<.001*** (7.03)^b^ |
| Rate and amount of speech (YMRS 6) | .29 (.92) | .2 (.68) | .21 (.76) | 1.02 (2.51) | p<.001*** (54.36)^b^ |
| Language-thought-disorder (YRMS 7) | .02 (.13) | 1.27 (1.85) | .04 (.2) | 1.1 (.9) | p<.001*** (312.81)^b^ |
| Retardation (HAMD 16) | .02 (.13) | .04 (.19) | 1.15 (.36) | .69 (.95) | p<.001*** (523.65)^f^ |

Note: Mean (standard deviation), SANS (scale for the assessment of negative symptoms), SAPS (scale for the assessment of positive symptoms), YMRS (Young Mania rating scale), HAMD (Hamilton rating scale for Depression), *** significant after correction for multiple testing (Benjamini Hochberg).

^a^ Cluster 1, 3 < Cluster 2, 4

^b^ Cluster 1, 2, 3 < Cluster 4

^c^ Cluster 1 < Cluster 2, 3, 4; Cluster 3 < Cluster 2, 4

^d^ Cluster 1, 2, 3 < Cluster 4; Cluster 2 < Cluster 4

^e^ Cluster 1 < Cluster 2, 4; Cluster 2, 3 < Cluster 4

^f^ Cluster 1 < Cluster 2, 3, 4; Cluster 2, 4 < Cluster 3

# eTable3: Demographic and clinical characteristics of latent clusters.

|  | **Cluster 1 (minimal FTD)** | **Cluster 2**  **(poverty)** | **Cluster 3**  **(inhibition)** | **Cluster 4**  **(severe FTD)** | **Group comparison  (F-value )** |
| --- | --- | --- | --- | --- | --- |
| Age | 36.98 (12.87) | 38.74 (12.4) | 36.91 (13.75) | 39.49 (11.86) | p=.359  (1.07) |
| Years of education | 13.38 (2.7) | 12.75 (3.04) | 13.04 (2.64) | 12.96 (2.79) | *p*=.167  (1.69) |
| YRMS sum | 1.46 (2.26) | 1.27 (1.85) | 1.52 (2.0) | 7.95 (8.35) | *p*<.001***  *(90.52)^a^* |
| HAMD sum | 6.6 (5.59) | 10.98 (7.17) | 12.41 (6.16) | 9.14 (6.52) | *p*<.001***  *(51.93)^b^* |
| SANS sum | 5.13 (6.33) | 17.24 (11.03) | 12.07 (9.06) | 15.24 (14.99) | *p*<.001***  (99.48)^c^ |
| SANS alogia | .21 (.74) | 3.24 (1.86) | .4 (.78) | 2.9 (3.42) | *p*<.001***  (238.14)^d^ |
| SANS affective flattening | 1.04 (2.54) | 5.33 (5.28) | 3.71 (4.48) | 4.1 (6.21) | p<.001***  (63.66)^e^ |
| SANS avolition/apathy | 1.32 (1.88) | 3.34 (2.89) | 2.48 (2.60) | 3.29 (3.04) | *p*<.001***  (41.10)^c^ |
| SANS anhedonia/asociality | 2.15 (3.03) | 4.0 (3.39) | 4.6 (3.98) | 3.68 (4.5) | *p*<.001***  (32.06)^f^ |
| SANS attention | .4 (.98) | 1.34 (1.86) | .84 (1.14) | 1.27 (1.85) | *p*<.001***  (24.75)^e^ |
| SAPS sum | .92 (3.01) | 2.35 (5.45) | .9 (1.94) | 12.71 (12.4) | *p*<.001***  (133.18)^g^ |
| SAPS positive formal thought disorder | .37 (1.30) | .73 (1.36) | .38 (1.29) | 6.41 (4.91) | *p*<.001***  (199.93)^f^ |
| SAPS hallucinations | .16 (1.09) | .63 (2.38) | .1 (.48) | 1.64 (3.96) | *p*<.001***  (22.05)^h^ |
| SAPS delusions | .34 (1.66) | .87 (2.65) | .32 (1.2) | 3.76 (6.45) | *p*<.001***  (41.44)^a^ |
| SAPS bizarre behaviour | .05 (.35) | .11 (.5) | .09 (.56) | .85 (1.76) | *p*<.001***  (39.08)^a^ |

^a^ Cluster 1, 2, 3 < Cluster 4

^b^ Cluster 1 < Cluster 1, 2, 3; Cluster 4 < Cluster 3

^c^ Cluster 1 < Cluster 2, 3, 4; Cluster 3 < Cluster 2, 4

^d^ Cluster 1 < Cluster 2, 4; Cluster 3 < Cluster 2, 4

^e^ Cluster 1 < Cluster 2, 3, 4; Cluster 3 < Cluster 2

^f^ Cluster 1 < Cluster 2, 3, 4

^g^ Cluster 1 < Cluster 2, 4; Cluster 2, 3 < Cluster 4

^h^ Cluster 1, 2, 3 < Cluster 4, Cluster 1, 3 < Cluster 2

Note: Mean (standard deviation), SANS (scale for the assessment of negative symptoms), SAPS (scale for the assessment of positive symptoms), YMRS (Young Mania rating scale), HAM-D (Hamilton rating scale for Depression), *** significant after correction for multiple testing (Benjamini Hochberg).

# eTable4: Neurocognitive characteristics of FTD clusters.

|  | **Cluster 1 (minimal FTD)** | **Cluster 2**  **(poverty)** | **Cluster 3**  **(inhibition)** | **Cluster 4**  **(severe FTD)** | **Group comparison  (F-value )** |
| --- | --- | --- | --- | --- | --- |
| Working memory  (letter-number-span) | 15.98 (3.23) | 14.04 (3.77) | 15.24 (3.39) | 13.80 (3.22) | *p*<.001***  (16.53)^a^ |
| Attention (D2) | 170.93 (42.33) | 149.47 (46.63) | 159.99 (43.51) | 140.49 (42.1) | *p*<.001***  (14.25)^b^ |
| Phonemic verbal fluency (RWT) | 11. 58 (4.16) | 9.84 (4.4) | 10.04 (4.26) | 8.9 (4.23) | *p*<.001***  (13.54)^c^ |
| Semantic verbal fluency (RWT) | 23.44 (5.85) | 20.06 (6.8) | 22.29 (5.79) | 20.51 (5.19) | *p*<.001***  (11.39)^d^ |
| Execution (TMT A) | 25.97 (9.67) | 32.35 (12.03) | 27.58 (9.51) | 33.64 (14.71) | *p*<.001***  (18.17)^e^ |
| Execution (TMT B) | 54.87 (21.51) | 68.58 (28.25) | 58.41 (21.83) | 76.05 (35.06) | *p*<.001***  (21.89)^e^ |
| Verbal episodic memory (CVLT) | 55.86 (9.71) | 51.82 (11.35) | 53.82 (10.02) | 48.31 (10.93) | *p*<.001***  (13.47)^d^ |

Note: Mean (standard deviation), RWT (Regensburger Wortflüssigkeitstest), TMT (Trail-Making-Test), CVLT (California Verbal Learning Test), *** significant after correction for multiple testing (Benjamini Hochberg).

^a^ Cluster 1, 3 > Cluster 2, 4

^b^ Cluster 1 > Cluster 2, 3, 4; Cluster 3 > Cluster 4

^c^ Cluster 1 > Cluster 2, 3, 4

^d^ Cluster 1 > Cluster 2, 4; Cluster 3 > Cluster 2

^e^ Cluster 1, 3 < Cluster 2, 4

| eTable5: Sample characteristics of the age- and sex-matched subsample with same n per diagnosis (N=321). | | | | |
| --- | --- | --- | --- | --- |
|  | **MDD**  (*n*=107) | **BD**  (*n*=107) | **SSD (SZ/SZA)**  (*n*=107) | **Group comparison**  (F-value/Chi in brackets) |
| Age | 38.06 (11.68) | 39.21 (11.22) | 38.07 (11.7) | *p*=.599  (.51) |
| Sex | 55 m / 52 f | 52 m / 55 f | 55 m / 52 f | *p*=.894  (.22) |
| Years of education | 12.86 (2.96) | 14.03 (2.78) | 12.46 (2.71) | *p*<.001^a^***  (8.53) |
| TIV | 1566.13  (177.22) | 1589.61  (141.67) | 1580.23  (188.16) | *p*=.552  (.59) |
| HAMD sum | 7.95 (6.14) | 6.53 (5.57) | 6.69 (5.79) | *p*=.195  (1.64) |
| YRMS sum | 1.14 (1.94) | 3.65 (5.58) | 2.41 (4.73) | *p*<.001^b^***  (8.84) |
| SAPS sum | .62 (1.58) | 2.52 (4.53) | 8.93 (11.48) | *p*<.001^c^***  (40.44) |
| SAPS positive formal thought disorder | .2 (.68) | 1.8 (3.32) | 2.79 (3.99) | *p*<.001^d^***  (18.82) |
| SAPS hallucinations | .16 (.66) | .19 (.85) | 1.88 (4.13) | *p*<.001^c^***  (19.06) |
| SAPS delusions | .24 (.87) | .32 (.88) | 3.94 (5.99) | *p*<.001^c^***  (40.71) |
| SANS sum | 6.82 (7.56) | 4.92 (6.66) | 12.81 (11.54) | *p*<.001^c^***  (18.29) |
| SANS alogia | .6 (1.3) | .54 (1.32) | 1.75 (2.6) | *p*<.001^c^***  (14.54) |
| SANS anhedonia | 2.41 (3.07) | 1.83 (2.98) | 2.78 (3.47) | *p*=.092  (2.4) |
| Mean (standard deviation), SANS (scale for the assessment of negative symptoms), SAPS (scale for the assessment of positive symptoms), YMRS (Young Mania rating scale, HAMD (Hamilton rating scale for Depression), TIV (Total intracranial volume), *** significant after correction for multiple testing (Benjamini Hochberg). | | | | |
| ^a^ MDD, SSD < BD  ^b^ MDD < BD  ^c^ MDD, BD < SSD  ^d^ MDD, BD < SSD, MDD < BD eTable6: Fit indices of the competing models (equal variance and covariance across clusters) in the age- and sex-matched subsample N=321.  \| **Number of clusters** \| **AIC** \| **BIC** \| **Entropy** \| **prob. min.** \| ***n* min.** \| **BLRT p-value** \| \| --- \| --- \| --- \| --- \| --- \| --- \| --- \| \| 2 \| 11868.31468 \| 12437.80229 \| .95958 \| .97561 \| .25234 \| .0099 \| \| 3 \| 11583.87637 \| 12213.70704 \| .96958 \| .98622 \| .14953 \| .0099 \| \| **4** \| **11491.69471** \| **12181.86843** \| **.95587** \| **.96982** \| **.09346** \| **.0099** \| \| 5 \| 11500.48933 \| 12251.00611 \| .96833 \| .92359 \| .03427 \| .58416 \| | | | | |

# eTable7: FTD psychopathological characteristics of latent clusters in an age- and sex-matched sample with same n per DSM-IV-TR diagnoses (N=321).

|  | **Cluster 1 (minimal FTD)** | **Cluster 2**  **(poverty)** | **Cluster 3**  **(inhibition)** | **Cluster4**  **(severe FTD)** | **Group comparison  (F-value )** |
| --- | --- | --- | --- | --- | --- |
| Poverty of speech (SANS 8) | .14 (.54) | .9 (1.12) | .17 (.54) | .38 (.89) | p=.003 (5.08) |
| Poverty of content of speech (SANS 9) | .07 (.32) | .33 (.55) | .12 (.39) | .69 (1.13) | p=.001 (6.64) |
| Blocking (SANS 10) | .04 (.24) | .17 (.53) | .02 (.16) | .48 (.89) | p=.006 (4.44) |
| Latency of response (SANS 11) | .09 (.32) | 2.27 (.64) | .22 (.42) | .71 (1.03) | p<.001 (113.99)*** |
| Derailment (SAPS 26) | .05 (.29) | .03 (.18) | 0 (0) | .77 (1.15) | p<.001 (115.21)*** |
| Tangentiality (SAPS 27) | .08 (.34) | .13 (.43) | .09 (.37) | 1.29 (1.39) | p<.001 (11.72)*** |
| Incoherence (SAPS 28) | .01 (.09) | .07 (.25) | 0 (0) | .42 (.87) | p<.001 (78.22)*** |
| Illogicality (SAPS 29) | .01 (.12) | .17 (.53) | 0 (0) | .48 (.79) | p<.001 (121.34)*** |
| Circumstantiality (SAPS 30) | .11 (.41) | .60 (.77) | .19 (.46) | 1.44 (1.30) | p<.001 (19.40)*** |
| Pressure of speech (SAPS 31) | .18 (.59) | .07 (.37) | .19 (.64) | 1.5 (1.57) | p<.001 (12.07)*** |
| Distractible speech (SAPS 32) | .01 (.09) | .07 (.25) | .12 (.33) | 1.48 (1.17) | p<.001 (26.66)*** |
| Clanging (SAPS 33) | .005 (.07) | 0 (0) | 0 (0) | .15 (.74) | p<.001 (37.32)*** |
| Rate and amount of speech (YMRS 6) | .34 (1.00) | .20 (.81) | .24 (.92) | 2.1 (2.32) | p<.001 (9.55)*** |
| Language-thought-disorder (YRMS 7) | .07 (.34) | .13 (.35) | .05 (.21) | .75 (.89) | p<.001 (9.54)*** |
| Retardation (HAMD 16) | .06 (.26) | .97 (.72) | 1.32 (.47) | .46 (.82) | p<.001 (105.25)*** |

Note: Mean (standard deviation), SANS (scale for the assessment of negative symptoms), SAPS (scale for the assessment of positive symptoms), YMRS (Young Mania rating scale), HAMD (Hamilton rating scale for Depression), *** significant after correction for multiple testing (Benjamini Hochberg).

# eFigure3: Four-cluster item profiles for FTD symptoms in the age- and sex-matched sample with same n per DSM-IV-TR diagnoses (N=321).


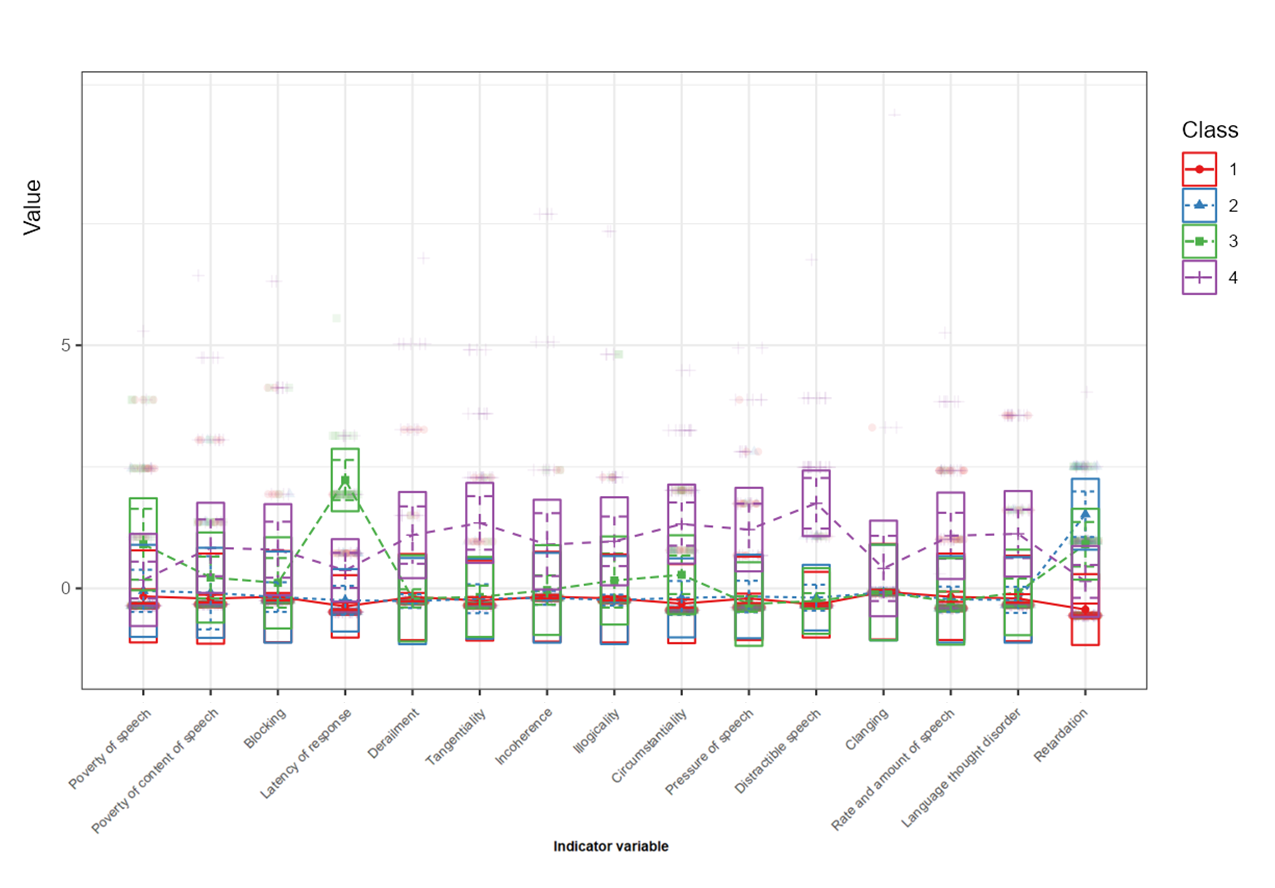


Note: Estimated standardized sample means of SANS, SAPS, YMRS and HAMD items used as indicator variables in the LPA across clusters. Boxes encompass +/− 1 SD. Cluster 1 (minimal overall FTD), cluster 2 (poverty), cluster 3 (inhibition), cluster 4 (severe overall FTD).

# eFigure4: Elbow plot for different cluster models in the age- and sex-matched sample with same n per DSM-IV-TR diagnoses (N=321).


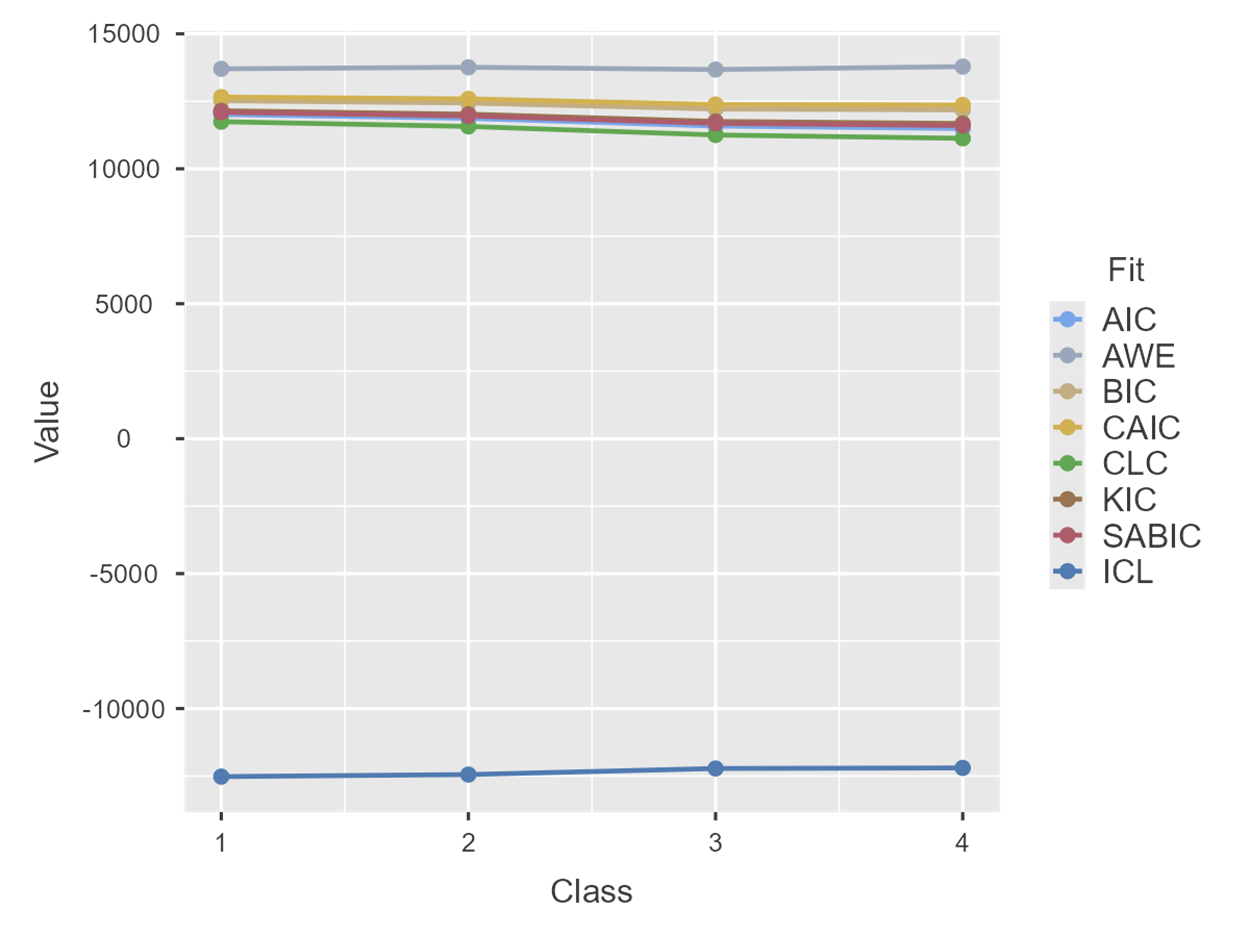


# eFigure5: Distribution of four-cluster item profiles for FTD symptoms in the age- and sex-matched subsample with same n per diagnosis (N=321).


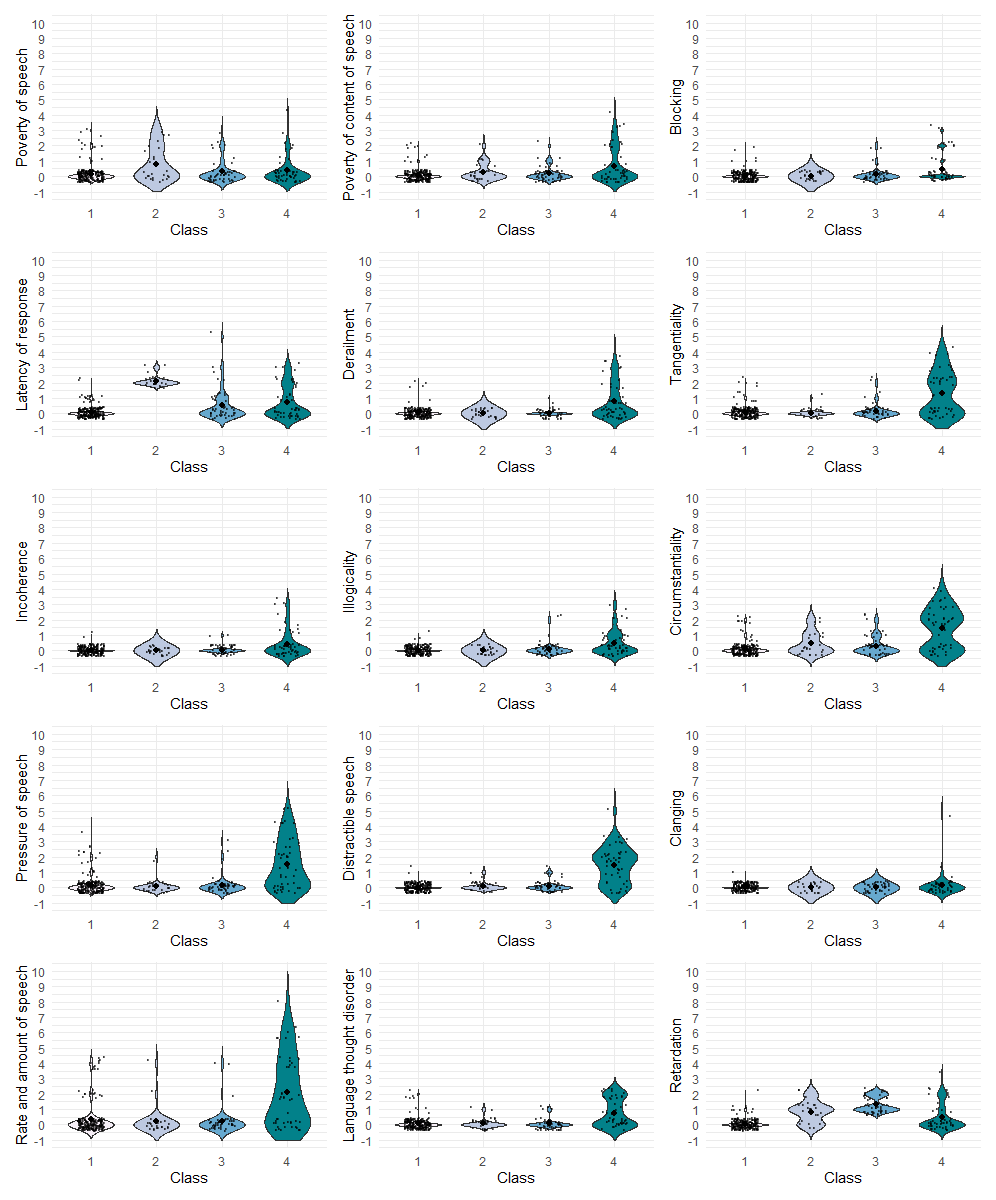


# eFigure6: Relative distribution of clinical diagnoses within latent FTD clusters in the age- and sex-matched subsample with same n per diagnosis (N=321).


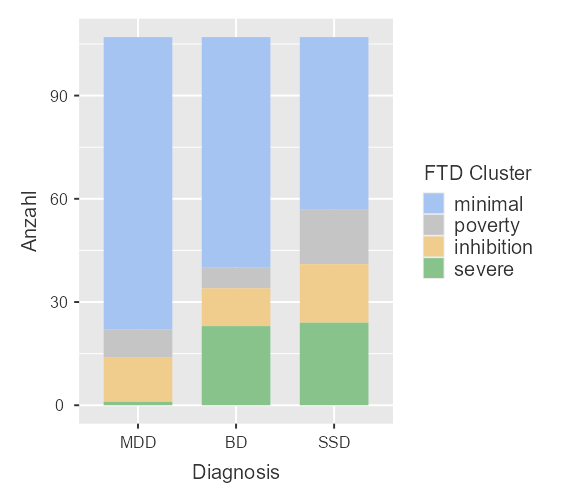


# eTable8: Fit indices of the competing models (equal variance and covariance across clusters) in MDD (n=800).

| **Number of clusters** | **AIC** | **BIC** | **Entropy** | **prob. min.** | ***n* min.** | **BLRT p-value** |
| --- | --- | --- | --- | --- | --- | --- |
| 2 | 27685.14120 | 28312.87917 | . 99949 | .99992 | .22875 | .0099 |
| 3 | 27498.17948 | 28196.18663 | .98715 | .99153 | .10250 | .0099 |
| **4** | **26601.59109** | **27369.86741** | **.99073** | **.99548** | **.07250** | **.0099** |
| 5 | 25660.72881 | 26499.2743.1 | .98886 | .98534 | .02375 | .0099 |

# eTable9: Fit indices of the competing models (equal variance and covariance across clusters) in BD (n=125).

| **Number of clusters** | **AIC** | **BIC** | **Entropy** | **prob. min.** | ***n* min.** | **BLRT p-value** |
| --- | --- | --- | --- | --- | --- | --- |
| 2 | 4836.76624 | 5263.84162 | .96376 | .98706 | .31200 | .0099 |
| 3 | 4764.31585 | 5236.64425 | .97294 | .96311 | .14400 | .0099 |
| **4** | **4725.65918** | **5243.24060** | **.98303** | **.98073** | **.08000** | **.0099** |
| 5 | 4629.39577 | 5192.23020 | .98618 | .97726 | .08400 | .0900 |

# eTable10: Fit indices of the competing models (equal variance and covariance across clusters) in SSD (n=107).

| **Number of clusters** | **AIC** | **BIC** | **Entropy** | **prob. min.** | ***n* min.** | **BLRT p-value** |
| --- | --- | --- | --- | --- | --- | --- |
| 2 | 4002.49686 | 4406.09402 | .98073 | .99353 | .45794 | .0099 |
| 3 | 3949.21284 | 4395.57525 | .97467 | .95938 | .11215 | .0099 |
| **4** | **3966.81160** | **4455.93928** | **.96150** | **.93781** | **.07477** | **.88119** |
| 5 | 3964.56958 | 4496.46251 | .91572 | .64233 | .07477 | .28713 |

# eTable11: Demographic and clinical characteristics of latent FTD clusters in the age- and sex-matched subsample with same n per diagnosis (N=321).

|  | **Cluster 1 (minimal FTD)** | **Cluster 2**  **(poverty)** | **Cluster 3**  **(inhibition)** | **Cluster 4**  **(severe FTD)** | **Group comparison  (F-value )** |
| --- | --- | --- | --- | --- | --- |
| Age | 38.90 (11.55) | 36.88 (11.78) | 36.70 (12.63) | 39.02 (10.13) | *p*=.664 (.53) |
| Years of education | 13.25 (2.97) | 12.80 (3.19) | 12.93 (2.67) | 12.96 (2.58) | *p*=.799 (.34) |
| YRMS sum | 1.48 (2.43) | .68 (1.18) | 1.54 (2.52) | 8.00 (8.15) | *p*<.001 (38.86) *** |
| HAMD sum | 5.75 (5.27) | 8.16 (5.03) | 11.26 (6.16) | 7.98 (6.26) | *p*<.001 (12.35) *** |
| SANS sum | 5.14 (6.75) | 15.96 (8.40) | 11.80 (7.71) | 11.79 (12.98) | *p*<.001 (23.02) *** |
| SANS alogia | .34 (103) | 3.20 (1.35) | 1.13 (2.14) | 2.25 (3.04) | *p*<.001 (38.79) *** |
| SANS anhedonia/asociality | 1.86 (2.91) | 3.40 (2.84) | 3.30 (3.27) | 2.90 (4.01) | *p*=.002 (4.89) |
| SAPS sum | 2.00 (5.77) | 2.84 (4.17) | 2.22 (3.43) | 14.75 (11.35) | *p*<.001 (47.40) *** |
| SAPS positive formal thought disorder | .46 (1.22) | .68 (.900) | .91 (1.71) | 7.52 (4.19) | *p*<.001 (152.54) *** |
| SAPS hallucinations | .55 (2.49) | .84 (2.82) | .36 (.96) | 1.83 (3.47) | *p*=.01 (3.86) |
| SAPS delusions | .92 (3.08) | 1.28 (2.17) | 1.00 (2.25) | 4.50 (6.75) | *p*<.001 (47.40) *** |

Note: Mean (standard deviation), SANS (scale for the assessment of negative symptoms), SAPS (scale for the assessment of positive symptoms), YMRS (Young Mania rating scale), HAM-D (Hamilton rating scale for Depression), *** significant after correction for multiple testing (Benjamini Hochberg).

# eTable12: Neurocognitive characteristics of FTD clusters in the age- and sex-matched subsample (N=321).

|  | **Cluster 1 (minimal FTD)** | **Cluster 2**  **(poverty)** | **Cluster 3**  **(inhibition)** | **Cluster 4**  **(severe FTD)** | **Group comparison  (F-value )** |
| --- | --- | --- | --- | --- | --- |
| Working memory  (letter-number-span) | 15.54 (3.44) | 13.10 (4.09) | 14.12 (3.39) | 13.67 (3.13) | *p*<.001 (7.34)*** |
| Attention (D2) | 164.06 (44.58) | 142.00 (51.48) | 148.37 (42.38) | 136.19 (35.28) | *p*<.001 (8.15)*** |
| Phonemic verbal fluency (RWT) | 10.82 (4.37) | 8.93 (4.88) | 9.56 (4.94) | 10.02 (5.13) | *p*=.114 (2.05) |
| Semantic verbal fluency (RWT) | 22.43 (5.58) | 19.83 (8.23) | 20.59 (5.54) | 20.44 (5.25) | *p*=.032 (3.09) |
| Execution (TMT A) | 27.69 (10.87) | 30.77 (12.53) | 29.09 (9.79) | 31.19 (10.68) | *p*=.011 (1.70) |
| Execution (TMT B) | 58.81 (24.63) | 75.03 (33.48) | 62.73 (24.08) | 70.73 (25.51) | *p*=.006 (4.45)*** |
| Verbal episodic memory (CVLT) | 53.22 (9.75) | 48.17 (11.23) | 50.85 (10.27) | 48.35 (11.18) | *p*=.010 (4.02) |

Note: Mean (standard deviation), RWT (Regensburger Wortflüssigkeitstest), TMT (Trail-Making-Test), CVLT (California Verbal Learning Test), *** significant after correction for multiple testing (Benjamini Hochberg).

# eTable 13: Differences between identified clusters in GMV and sulcal depth in the age- and sex-matched subsample (N=321).

|  | **H** | **x** | **y** | **z** | ***F/t*** | ***k*** | ***p*<.05** |
| --- | --- | --- | --- | --- | --- | --- | --- |
| **GMV** | | | | | | | |
| **cluster 1 (minimal overall FTD) > cluster 4 (severe overall FTD)** | | | | | | | |
| 61.3% superior frontal gyrus medial segment  38.7% anterior cingulate gyrus | R | 4 | 36 | 32 | 1.74 | 5 | .040 |
| **cluster 3 (inhibition) > cluster 1 (minimal overall FTD)** | | | | | | | |
| 46.5% inferior temporal gyrus  44.5% fusiform gyrus  9% temporal pole | R | 42 | -6 | -46 | 3.15 | 231 | .001 |
| 88.3% inferior temporal gyrus  10% middle temporal gyrus  2.7% fusiform gyrus | R | 51 | -21 | -22 | 2.14 | 16 | .016 |
| 34.1% middle temporal gyrus  18.6% temporal pole  4.5% inferior temporal gyrus  2.9% superior temporal gyrus | R | 50 | -2 | -30 | 1.87 | 13 | .031 |
| **cluster 3 (inhibition) > cluster 4 (severe overall FTD)** | | | | | | | |
| 88.7% inferior temporal gyrus  8% middle temporal gyrus  3.3 % fusiform gyrus | R | 51 | -22 | -22 | 3.84 | 1215 | **<.0001** |
| 75.7% middle temporal gyrus  17.1% temporal pole  4.8% superior temporal gyrus  2.3% inferior temporal gyrus | R | 50 | 2 | -28 | 3.19 | 177 | **.001** |
| **Sucal depth** | | | | | | | |
| **cluster 3 (inhibition) > cluster 2 (poverty)** | | | | | | | |
| \| 45% insula  37% superior temporal sulcus  16% temporal pole \| L \| -37 \| 3 \| -24 \| 2.02 \| 91 \| .02 \| \| --- \| --- \| --- \| --- \| --- \| --- \| --- \| --- \| | | | | | | | |
| **cluster 3 (inhibition) > cluster 4 (severe overall FTD)** | | | | | | | |
| \| 100% Insula \| R \| 38 \| -15 \| -7 \| 1.87 \| 287 \| .031 \| \| --- \| --- \| --- \| --- \| --- \| --- \| --- \| --- \| | | | | | | | |

*Note*: ROI analyses to test whether brain structural correlates can be replicated in the matched sample at p<.05 uncorrected. R=right, L=left, H=hemisphere; k=cluster extend; bold letters indicate significance after correction for multiple testing (Benjamini Hochberg).

# References

1. Kurth F, Luders E. Voxel-Based Morphometry. Brain Mapping: An Encyclopedic Reference. 2015;1:345–349.

2. Ashburner J, Friston KJ. Unified segmentation. Neuroimage. 2005;26:839–851.

3. Rajapakse JC, Giedd JN, Rapoport JL. Statistical approach to segmentation of single-channel cerebral MR images. IEEE Trans Med Imaging. 1997;16:176–186.

4. Tohka J, Zijdenbos A, Evans A. Fast and robust parameter estimation for statistical partial volume models in brain MRI. Neuroimage. 2004;23:84–97.

5. Ashburner J. A fast diffeomorphic image registration algorithm. Neuroimage. 2007;38:95–113.

6. Ashburner J, Neelin P, Collins DL, Evans A, Friston K. Incorporating prior knowledge into image registration. Neuroimage. 1997;6:344–352.

7. Dahnke R, Yotter RA, Gaser C. Cortical thickness and central surface estimation. Neuroimage. 2013;65:336–348.

8. Yotter RA, Dahnke R, Thompson PM, Gaser C. Topological correction of brain surface meshes using spherical harmonics. Hum Brain Mapp. 2011;32:1109–1124.

9. Yotter RA, Thompson PM, Gaser C. Algorithms to improve the reparameterization of spherical mappings of brain surface meshes. J Neuroimaging. 2011;21.
